# Efficacy of a Mobile Health–Based Behavioral Treatment for Lifestyle Modification in Type 2 Diabetes Self-Management: Greenhabit Randomized Controlled Trial

**DOI:** 10.2196/58319

**Published:** 2025-01-22

**Authors:** Ana Maria Ruiz-Leon, Rosa Casas, Sara Castro-Barquero, Sofia Alfaro-González, Petia Radeva, Emilio Sacanella, Francesc Casanovas-Garriga, Ainhoa Pérez-Gesalí, Ramon Estruch

**Affiliations:** 1 Department of Internal Medicine, Hospital Clinic, Institut d’Investigacio Biomèdica August Pi i Sunyer Barcelona Spain; 2 CIBERobn Fisiopatología de la Obesidad y la Nutrición, Instituto de Salud Carlos III Madrid Spain; 3 Institut de Recerca en Nutrició i Seguretat Alimentaria. University of Barcelona Barcelona Spain; 4 Fundación Dieta Mediterránea Barcelona Spain; 5 Faculty of Medicine and Life Sciences, University of Barcelona Barcelona Spain; 6 Faculty of Mathematics and Computer Science, University of Barcelona Barcelona Spain

**Keywords:** type 2 diabetes, cardiovascular health, lifestyle intervention, mHealth, artificial intelligence, mobile health, mobile application, diabetes, DM, self-management, randomized controlled trial, RCT, health care, self-care, Greenhabit, behavioral health, treatment, medication, analysis of covariance, intervention, work-life balance, cardiovascular, cardiovascular risk

## Abstract

**Background:**

Enhancing self-management in health care through digital tools is a promising strategy to empower patients with type 2 diabetes (T2D) to improve self-care.

**Objective:**

This study evaluates whether the Greenhabit (mobile health [mHealth]) behavioral treatment enhances T2D outcomes compared with standard care.

**Methods:**

A 12-week, parallel, single-blind randomized controlled trial was conducted with 123 participants (62/123, 50%, female; mean age 58.25 years, SD 9.46 years) recently diagnosed with T2D. Participants were recruited face-to-face from primary care centers in Barcelona, Spain, between July 2021 and March 2022. They were randomly assigned to 1 of 2 groups: (1) an intervention group (n=61) instructed to use the Greenhabit mobile app alongside standard care, or (2) a control group (n=62) who received advice on maintaining a healthy diet and followed standard care. The Greenhabit app incorporates serious gaming technology. Participants received daily messages and challenges focused on promoting a healthy lifestyle, including nutrition, exercise, relaxation, a positive mindset, and a supportive social environment. The app encouraged participants to set weekly goals and awarded points for completing challenges. Data on nutrition, anthropometrics, and blood and urine samples were collected at baseline, 6 weeks, and 12 weeks. Questionnaires assessing quality of life, work-life balance, and social environment were administered at baseline and during the final visit. The primary outcomes were HbA1c and fasting plasma glucose (FPG). Repeated-measures analysis of variance was used to compare changes over time (baseline to 6 weeks and baseline to 12 weeks) between the 2 intervention groups. Analysis of covariance was performed to evaluate changes at 6 and 12 weeks, adjusted for baseline levels of each variable. Multiple contrasts were corrected using a Bonferroni post hoc test.

**Results:**

Both groups showed significant reductions in HbA1c after 6 and 12 weeks (mean change in the intervention group [n=50] –0.4%, *P*<.001 vs –0.3% in the control group [n=53], *P*=.001) and in FPG after 6 weeks (mean change in the intervention group –5.3 mg/dL, *P*=.01 vs control group –5.8 mg/dL, *P*=.01). At 12 weeks, the intervention group also showed significant reductions in systolic and diastolic blood pressures (mean change –4.5, *P*=.049 and –2.4 mmHg, *P*=.03, respectively), body weight (mean change –0.8 kg, *P*=.03), BMI (mean change –0.3 kg/m2, *P*=.03), waist circumference (mean change –1.0 cm, *P*=.046), and triglyceride concentration (mean change –20.0 mg/dL, *P*=.03). There was also a significant increase in high-density lipoprotein-cholesterol concentrations (mean change 2 mg/dL, *P*=.049). Finally, improvements were noted in 3 out of the 5 elements of balance: positivity, social environment, and work-life balance.

**Conclusions:**

The 12-week intervention with the Greenhabit behavioral treatment mHealth app showed beneficial effects on T2D outcomes and reduced the burden of cardiovascular risk factors. Although larger studies are warranted, these results suggest that mHealth apps can be a promising tool for improving T2D self-management.

**Trial Registration:**

ISRCTN Registry ISRCTN13456652; http://www.isrctn.com/ISRCTN13456652

## Introduction

Diabetes is a chronic metabolic disease characterized by hyperglycemia, which results from defects in insulin secretion, insulin action, or both [1,2]. Over time, this condition leads to severe complications, contributing to significant morbidity and mortality worldwide [3].

According to the World Health Organization, diabetes remains a critical public health issue, with 422 million people affected in 2014. The global prevalence of diabetes is steadily increasing, with projections estimating 570 million cases by 2030 and 700 million by 2045, largely driven by rising rates of overweight and obesity [4]. The growing prevalence of type 2 diabetes (T2D) and the expanding population affected underscore an urgent need to develop novel strategies for diabetes treatment, treatment adherence, lifestyle modifications, and care. The rising prevalence of T2D places a significant strain on health care systems, highlighting the importance of empowering individuals to manage their conditions effectively [5]. Consequently, numerous mobile health (mHealth) apps have been developed as innovative tools to enhance self-management of T2D and prevent macrovascular and microvascular complications [6-12].

Patients with T2D require effective diabetes control and self-management skills to adopt a healthier lifestyle. Self-management encompasses daily activities aimed at controlling or reducing the disease’s impact on health and well-being, including monitoring blood glucose levels, managing food intake, engaging in physical activity, and managing stress [13]. mHealth apps play a pivotal role in achieving glycemic control, encouraging lifestyle modifications, and promoting self-management among individuals with T2D [14]. Recent research highlights the growing adoption of these apps for managing treatment and care among this population [13]. They empower self-management by supporting dietary adherence, consistent blood sugar monitoring, regular physical activity, reduced medication needs, and lower glycated hemoglobin (HbA_1c_) levels [15,16]. Additionally, they help reduce face-to-face counseling time and health care expenses [17].

Currently, a wide range of mHealth apps is available to enhance self-management for individuals with diabetes. These apps facilitate close monitoring, provide feedback, and help overcome geographic barriers [14]. Furthermore, a systematic review and meta-analysis have demonstrated the effectiveness of mHealth apps in empowering self-management across various domains of disease management and lifestyle changes [18]. Additionally, the American Diabetes Association guidelines advocate for the use of mHealth apps as a valuable tool for achieving effective lifestyle changes in diabetes prevention [19]. However, the lack of consensus on the definition of self-management interventions for chronic diseases [20,21], combined with diverse outcome measures—such as self-efficacy, health-related quality of life, activity participation, performance of daily living activities, depression, and self-confidence [22]—poses challenges in comparing interventions and interpreting results. Greenhabit is a holistic education program that empowers individuals to take control of their health by fostering sustainable behavior change. Its approach, which addresses the physical, mental, and social aspects of well-being, promotes mental resilience and self-awareness [23]. The Greenhabit method is grounded in 2 key theories: (1) the *McClelland Iceberg model,* which serves as a framework for building content, challenges, and measures for users [24]; and (2) the *Behavioral Change Wheel,* which informs the development of interventions and techniques within Greenhabit [25]. Social integration (through buddies and community) provides motivation and social support to help maintain healthy habits. It encourages individuals to take care of their health by offering nudges related to nutrition, physical activity, and social interactions, all aimed at promoting overall well-being. The game/app incorporates serious gaming technology. The vitality education program is a 12-week journey in which players focus on the 5 elements of Greenhabit: nutrition, exercise, relaxation, positive mindset, and social environment. It leverages the power of brain-based learning through methods such as nudges, content, challenges, rewards, working with a buddy, and engaging the senses with joy. The brain is plastic, allowing us to learn new habits. It takes at least 68 days to establish new habits [26]. After 12 weeks, players report increased awareness, resilience, and energy.

Thus, the primary aim of this study was to assess the efficacy of the Greenhabit app on T2D outcomes, specifically plasma glucose concentrations and HbA_1c_ levels, in patients with a recent diabetes diagnosis. As a secondary aim, we evaluated whether participation in the app led to improvements in blood pressure (BP), plasma lipids, adiposity parameters, and the maintenance of a healthy lifestyle after 12 weeks of use.

## Methods

### Study Design

This study is a 12-week, parallel-group, single-blind, single-center, randomized controlled trial (RCT) designed to assess the efficacy of the Greenhabit method on diabetes treatment and management. The intervention, a low-intensity self-management program, focuses on behavior change and incorporates evidence-based approaches to diabetes self-management education and support [27]. The diabetes specialist dietitian and nurse applied principles of motivational interviewing, including a person-to-person, client-centered approach aimed at eliciting behavior change by helping patients explore and resolve ambivalence. Additionally, a successful telephonic intervention, proven to improve diabetes control in urban adults, was incorporated.

Consecutive candidates were randomly assigned to either the intervention or control group after sex stratification, with an allocation ratio of 1:1.

### Outcomes

The primary outcome of the trial was the management of T2D, measured by HbA_1c_ and fasting plasma glucose (FPG) concentrations at 12 weeks. The secondary outcomes included the effect of the intervention on cardiovascular risk factors, such as BP, lipid profile, body weight (BW), and adiposity, as well as its impact on dietary habits, stress relief, and psychological factors, assessed using validated questionnaires.

### Setting and Participants

**Figure 1:**
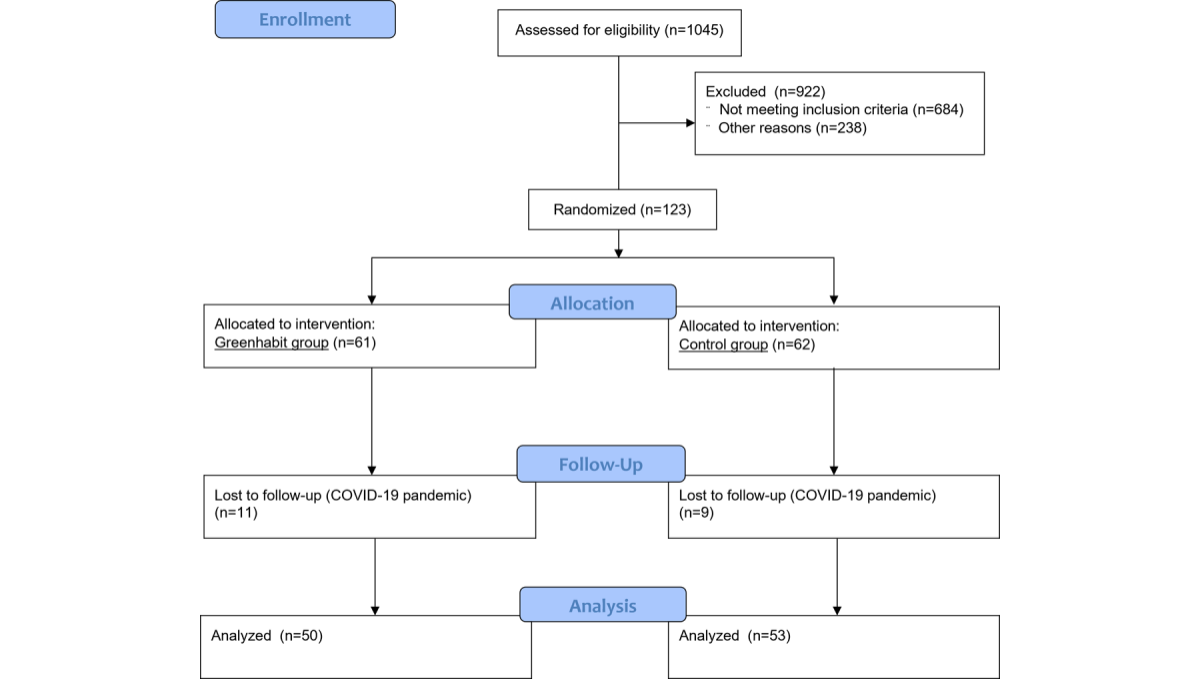
CONSORT (Consolidated Standards of Reporting Trials) flow diagram of the Greenhabit 12-week randomized controlled trial.

As shown in Figure 1, from July 2021 to March 2022, we screened 1045 potential candidates from the databases of primary care centers affiliated with Hospital Clínic of Barcelona, Spain. Of the screened participants, 684 were excluded for not meeting the inclusion criteria of a recent T2D diagnosis, while 238 were excluded for various other reasons: 179 had limited ability to use a smartphone, 48 had a severe chronic disease, 5 faced language barriers, 4 lacked a smartphone, and 2 had difficulty following an appropriate diet. Finally, 123 participants were recruited through a face-to-face interview and randomized, with 61 assigned to the Greenhabit group and 62 to the control group. Randomization was carried out by consecutively assigning participants using sealed envelopes, which were prepared based on a computer-generated random number table. Of these participants, 20 were withdrawn from the trial because they could not attend follow-up visits during the COVID-19 pandemic. As a result, 50 participants in the intervention group and 53 in the control group completed the study.

Inclusion criteria were as follows: men and women aged 18-75 years, with a recent diagnosis of T2D (<2 years of treatment with insulin, antidiabetic medications, a combination of both, or a nonpharmacological treatment), and either an HbA_1c_ greater than 6.5%, FPG greater than 126 mg/dL on 2 occasions, or a 2-hour plasma glucose greater than 200 mg/dL after a 75-g oral glucose load.

Exclusion criteria included participants who underwent or planned bariatric surgery during the trial period; individuals with type 1 diabetes; those with a diagnosis of gestational diabetes; and individuals with severe chronic illnesses, alcoholism, or other drug addictions. Also excluded were participants with gastrointestinal diseases that would hinder adherence to a specific diet, those who had taken vitamins or nutritional supplements in the month before recruitment, pregnant women, individuals without their own smartphone, those unable to attend additional visits, and those already participating in another trial.

### Ethics Approval and Consent to Participate

Before starting the study, all participants provided informed consent during a face-to-face interview with the researcher. The study protocol and procedures were approved by the Committee for Ethical Research at Hospital Clínic (Barcelona, Spain) on April 19, 2021, and adhere to the ethical standards of the Declaration of Helsinki. The study was registered with the following code: HCB/2021/0061. The trial was also registered with the International Standard Randomized Controlled Trial Number registry (ISRCTN13456652).

### Intervention

Participants in the intervention group were instructed to follow standard care recommendations, similar to the control group, and were provided with personalized, free access to the Greenhabit app via email. Additionally, they received a second free access for a partner (eg, sibling, friend, spouse) to offer extra motivation and mutual support. Participants could choose whether or not to use this second access. Each week, 3-4 days before starting the Greenhabit intervention, participants, grouped into teams of 10-12 individuals, were invited to attend an online group meeting. The purpose of these meetings was to teach participants how to use the app and address any potential technological barriers. Participants could invite friends or colleagues to join their journey for free; these invited individuals could only view the photos selected by the participants. Additionally, participants were encouraged to set weekly goals and log their measurements (eg, steps walked, weight, glucose levels, HbA_1c_). Each day, participants received a “treasure chest” from Greenhabit, containing messages and challenges related to healthy lifestyle habits (nutrition, exercise, relaxation, positive thinking, and social environment). Participants earned points for each challenge they completed. Various rewards, such as a water bottle for tracking intake, a jump rope, a notebook, and card games, were given throughout the 12-week program upon completing each level.

Participants in the control group received guidance on maintaining a healthy diet and were given written materials prepared by a dietitian. These materials included information on seasonal Mediterranean foods, shopping lists, weekly meal plans, and recipes for a typical week. Additionally, participants in this group received standard care through their primary care center. Usual care included routine health care appointments with providers, as well as access to diabetes management and weight loss education, electronic learning materials, and guidance on how to use the health system’s patient portal [28]. The main goal was to equip participants with the skills needed to maintain or improve their health.

### Greenhabit Behavioral Treatment Program

The Greenhabit program is a 12-week mHealth cognitive-behavioral education initiative designed specifically to support individuals in managing T2D. Greenhabit is a behavioral change program in which patients receive personalized content (interventions) based on their clinical profile through a recommender system. This system leverages an artificial intelligence–based infrastructure to promote personalized lifestyle changes. Additional information about the questionnaires used can be found in Supplementary Methods S1 in Multimedia Appendix 1 (see also [23-25,29-34]).

### Diet and Clinical Measurements

All participants underwent a face-to-face interview with the dietitian at baseline and after 12 weeks of intervention, along with regular phone calls. During these individual sessions, each participant received information on seasonal foods, shopping lists, weekly meal plans, and cooking recipes. Additionally, the individual interviews included a 151-item validated Food Frequency Questionnaire [35] and personalized recommendations for dietary changes aimed at achieving the participant’s goals. Participants also completed a 47-item questionnaire on education, lifestyle, medical history, and medication use. To assess adherence to the Mediterranean diet, a validated 14-point Mediterranean diet screener was used [36]. In the intervention group, participants were encouraged to increase their intake of vegetables (≥2 servings/day), fresh fruit (≥3 servings/day), legumes, nuts, and fish or seafood (≥3 servings/week), and to use olive oil for cooking and dressings. The app also promoted physical activity. Energy restriction was not specifically advised for either group.

At baseline (visit 1), 6 weeks (visit 2), and 12 weeks (visit 3), trained personnel (consistently the same dietitian and nurse) collected anthropometric measurements, determined BP, and took biological samples. Height was measured to the nearest 0.1 cm using a wall-mounted stadiometer with participants standing, wearing socks, and with their heads in the horizontal plane. BW was measured to the nearest 0.1 kg, with participants in light clothing and without shoes or accessories, using a high-quality calibrated scale. BMI was calculated by dividing weight (kg) by the square of height (m^2^). Waist circumference (WC) was measured midway between the lowest rib and the iliac crest using an anthropometric tape. Hip circumference (HC) was measured at the maximum point below the waist, without compressing the skin [37]. BP was measured in triplicate after at least 5 minutes of rest using a validated semiautomatic oscillometer (Omron HEM-705CP). The mean value of the 3 BP measurements was used in this study. A medical record was also collected. Fasting blood and spot urine samples were obtained for basic hematologic and biochemical analyses for all participants, including HbA_1c_, glucose, and lipid profile parameters. Additionally, the nurse provided diabetes self-management support to patients as needed.

Technicians and nurses were blinded to the group assignment of each participant.

### Quality-of-Life Questionnaires

At baseline and after 12 weeks, participants completed several questionnaires to assess perceived physical and mental health over time, focusing on various aspects of health-related quality of life, including energy, happiness, positivity, social environment, and work-life balance. Additional details about the tests used can be found in Supplementary Methods S1 in Multimedia Appendix 1.

### Validity and Reliability

Blood samples were collected at baseline and at the end of the intervention to determine biochemical and nutritional parameters. Hemoglobin was measured by high-performance liquid chromatography, blood glucose by the glucose oxidase method, and total cholesterol and triglycerides by enzymatic procedures. High-density lipoprotein (HDL) cholesterol was assessed after precipitation with phosphotungstic acid and magnesium chloride. All analyses were performed using the Advia 2400 clinical chemistry analyzer (Siemens Healthcare), with reagents provided by the instrument manufacturer. The main outcomes of the trial (HbA_1c_ and FPG levels) were determined at the CORE Laboratory at Hospital Clinic de Barcelona, which meets all required quality standards and holds ISO 9001:2015 certification and ISO 15189 accreditation for certain tests.

The instruments used for dietary assessment are both validated and have been utilized in large randomized clinical trials and cohort studies (see [35,36]).

Regarding quality of life, all the questionnaires are detailed in Supplementary Methods S1 in Multimedia Appendix 1. These questionnaires are divided into 5 components: energy, happiness, positivity, social life, and work-life balance. To assess these components, we used validated questionnaires tailored to the Spanish population, and further details of these questionnaires are extensively presented in Multimedia Appendix 1. In brief, the International Physical Activity Questionnaire, in its Spanish version, was used to assess physical activity [38,39]. The 36-item Short Form Health Survey was used to measure quality of life. This generic scale is applicable to both patients and the general population, providing a comprehensive profile of health status. The questionnaire includes 8 scales: Physical Function, Physical Role, Body Pain, General Health, Vitality, Social Function, Emotional Role, and Mental Health. The Spanish version is a validated instrument, suitable for both medical research and clinical practice [40]. The Hospital Anxiety and Depression Scale is a validated, rapid self-assessment screening tool. Although not intended for diagnostic purposes, it is highly convenient for evaluating anxiety and depression in patients with somatic or mental health issues [41]. This test has been widely used in patients with T2D [42]. Additionally, the Life Orientation Test assesses the psychometric properties of an optimism scale and has been adapted for the Spanish population using the multidimensional graded response model. This 10-item scale evaluates an individual’s life expectations, including 3 positive and 3 negative items. The test has also been widely used in individuals with T2D [43]. Finally, the Duke-UNK Functional Social Support Questionnaire is an objective tool that measures “perceived functional social support.” It is a self-administered, validated instrument for the Spanish population [44].

### Statistical Analysis

The total number of participants included was 123. Power calculations, based on an estimated intervention-induced intergroup difference of 0.8% in HbA_1c_ with an SD of 2.75, indicated that 104 individuals were needed to detect a statistically significant difference of .05 units (α risk=.05 and β risk=.2 in a bilateral test). A follow-up loss rate of 10% was anticipated. A reduction in HbA_1c_ greater than 0.5% is considered clinically significant, as diabetes-related complications are directly proportional to blood HbA_1c_ levels [45].

We used descriptive statistics, presenting the mean (SD) for the baseline characteristics of the participants. Categorical variables are expressed as percentages. For baseline characteristics, the Pearson chi-square test was used for categorical variables, and 1-way analysis of variance (ANOVA) was applied for continuous variables to compare between groups. Differences in biochemical parameters, anthropometric measures, food, and nutrient intake at baseline, 6 weeks, and 12 weeks of intervention were assessed using ANOVA. Changes in biochemical parameters and anthropometric variables after 6 and 12 weeks of intervention were assessed using analysis of covariance (ANCOVA), adjusting for baseline levels of each variable, age, sex, use of hypoglycemic treatment (yes/no), and BMI. BMI was categorized according to World Health Organization classifications: normal weight (<25 kg/m^2^), overweight (25 to <30 kg/m^2^), obesity class 1 (30 to <35 kg/m^2^), obesity class 2 (35 to <40 kg/m^2^), and obesity class 3 (≥40 kg/m^2^). Significant interactions were analyzed using simple-effect analysis, and multiple contrasts were adjusted using a Bonferroni post hoc test. Within- and between-group differences were expressed as estimated means with 95% CIs. The significance level was set at *P*<.05. All analyses were conducted using SPSS version 20.0 (IBM Corp.).

## Results

### Study Population

Of the 123 participants assessed (Figure 1; Multimedia Appendix 2), 31 in the Greenhabit group (n=61, 51%) and 31 in the control group (n=62, 50%) were female. The mean BMI was 31.8 (SD 6.14) kg/m^2^ for the Greenhabit group and 32.4 (SD 6.49) kg/m^2^ for the control group. Participants in the Greenhabit group were slightly younger, with a mean age of 56.0 (SD 10.8) years, compared with 60.5 (SD 8.11) years in the control group. No significant between-group differences were observed (Table 1).

**Table 1:** Baseline characteristics of the participants in the 2 study groups of the Greenhabit 12-week randomized controlled trial.

Variables			Greenhabit (n=61)		Control (n=62)		*P* value^a^
Age (years), mean (SD)			56.0 (10.8)		60.5 (8.11)		.01
Women, n (%)			31 (51)		31 (50)		.93
**Smoking status, n (%)**							
	Current smokers	18 (30)		11 (18)		.15	
**Educational level, n (%)**							.16
	Primary	14 (23)		23 (37)			
	Secondary	18 (30)		17 (27)			
	Higher	29 (48)		19 (31)			
**Marital status, n (%)**							.55
	Married	35 (57)		42 (68)			
	Single	11 (18)		9 (15)			
	Divorced, separated, or widowed	15 (25)		11 (18)			
**Alcohol drinker’s status, n (%)**							
	Current alcohol drinkers	45 (74)		39 (63)		.36	
MedDiet P14-score^b^ (points), mean (SD)			8.25 (2.17)		8.26 (1.92)		.80
**Adherence MedDiet^b^, n (%)**							.55
	Low adherence (<6 points)	6 (10)		3 (5)			
	Medium adherence (6-9 points)	36 (59)		40 (65)			
	High adherence (≥10 points)	19 (31)		19 (31)			
Body weight (kg), mean (SD)			87.6 (20.5)		88.1 (21.6)		.34
BMI (kg/m^2^), mean (SD)			31.8 (6.14)		32.4 (6.49)		.60
**BMI, n (%)**							.09
	18.5-24.9	4 (7)		10 (16)			
	25-29.9	22 (36)		16 (26)			
	30-34.9	14 (23)		21 (34)			
	35-39.9	16 (26)		8 (13)			
	≥40	5 (8)		7 (11)			
Waist circumference (cm), mean (SD)			106.7 (13.6)		108.2 (15.2)		.55
Hip circumference (cm), mean (SD)			110.8 (13.3)		113.0 (15.8)		.42
Waist-to-height ratio, mean (SD)			0.67 (0.08)		0.68 (0.09)		.47
Systolic blood pressure (mmHg), mean (SD)			141.2 (18.5)		144.1 (19.2)		.42
Diastolic blood pressure (mmHg), mean (SD)			84.8 (9.82)		81.8 (9.95)		.11
Glucose (mg/dL), mean (SD)			130.1 (33.3)		134.9 (38.2)		.49
Glycated hemoglobin (%), mean (SD)			6.91 (1.19)		6.96 (0.78)		.82
**Medications, n (%)**							
	Oral hypoglycemic drugs	45 (74)		44 (71)		.84	
	No antidiabetic medication	13 (21)		18 (29)		.85	
	Antihypertensive agents	23 (38)		20 (32)		.53	
	Lipid-lowering agents	20 (33)		28 (45)		.14	

^a^From the Pearson chi-square test for categorical variables and 1-factor analysis of variance for continuous variables.

^b^MedDiet P14-score: Mediterranean diet adherence evaluated using a 14-point score.

No changes in medication (glucose-lowering agents) were reported after the 12-week intervention, and no comorbidities associated with T2D were observed.

### Blood Pressure, Cardiovascular Risk Factors, and Adiposity

Table 2 (adjusted variables) and Table S1 in Multimedia Appendix 1 (unadjusted variables) present baseline values and changes in anthropometric measures and cardiovascular risk factors at 6 and 12 weeks by the intervention group. Participants in both groups showed reductions in systolic BP at 12 weeks (*P*=.049 and *P*=.02 in the intervention and control groups, respectively), FPG at 6 weeks, and HbA_1c_ at both 6 and 12 weeks (*P<*.001 for both). At 6 weeks, participants in the intervention group demonstrated a significant improvement in diastolic BP (*P=*.007). By 12 weeks, they also showed significant reductions in triglyceride concentrations (*P=*.03), BW (*P*=.03), BMI (*P*=.03), WC (*P*=.046), and HC (*P=*.049), along with a significant increase in HDL-cholesterol levels (*P*=.049).

**Table 2:** Changes in blood pressure, cardiovascular risk factors, and adiposity, at baseline and after 6 and 12 weeks of follow-up in the 2 study groups of the Greenhabit randomized controlled trial^a^.

Variables		Greenhabit (n=50)	Control (n=53)	Differences between groups (Greenhabit vs control)
**Systolic blood pressure (mmHg)**				
	Baseline	141.3 (19.5)	142.5 (18.5)	N/A^b^
	Changes after 6 weeks	–3.7 (–8.0 to 0.5)	–1.8 (–5.9 to 2.3)	4.0 (–7.6 to 15.6)
	Changes after 12 weeks	–4.5 (–9.0 to 0.0)^c^	–5.0 (–9.4 to –0.7)^c^	1.3 (–10.2 to 12.8)
**Diastolic blood pressure (mmHg)**				
	Baseline	84.9 (10.6)	81.2 (10.1)	N/A
	Changes after 6 weeks	–2.9 (–5.0 to –0.8)^c^	0.5 (–1.5 to 2.5)	–6.3 (–11.3 to –1.3)^d^
	Changes after 12 weeks	–2.4 (–4.4 to –0.3)^c^	–0.4 (–2.5 to 1.6)	–3.5 (–8.9 to 1.8)
**Fasting plasma glucose (mg/dL**)				
	Baseline	125.7 (29.1)	132.4 (38.0)	N/A
	Changes after 6 weeks	–5.3 (–9.6 to –1.0)^c^	–5.8 (–10.1 to –1.5)^c^	–0.09 (–9.0 to 8.8)
	Changes after 12 weeks	–4.0 (–9.3 to 1.2)	–1.4 (–6.5 to 3.6)	–2.7 (–14.3 to 8.9)
**Glycated hemoglobin** **(%)**				
	Baseline	6.88 (1.12)	6.90 (0.76)	N/A
	Changes after 6 weeks	–0.4 (–0.6 to –0.3)^e^	–0.3 (–0.4 to –0.1)^e^	–0.1 (–0.5 to –0.2)
	Changes after 12 weeks	–0.4 (–0.5 to –0.2)^e^	–0.3 (–0.4 to –0.1)^c^	–0.1 (–0.6 to –0.3)
**Triglycerides (mg/dL**)				
	Baseline	157.3 (65)	143.1 (55.2)	N/A
	Changes after 6 weeks	–12.1 (–25.4 to 1.1)	–2.4 (–15.3 to 10.6)	12.3 (–28.0 to 52.6)
	Changes after 12 weeks	–20.0 (–35.3 to –2.7)^c^	–7.8 (–23.9 to 8.3)	–14.1 (–73.8 to 45.6)
**Total cholesterol (mg/dL**)				
	Baseline	177.0 (40.7)	181.3 (36.3)	N/A
	Changes after 6 weeks	0.4 (–5.8 to 6.6)	1.3 (–4.8 to 7.4)	4.1 (–8.8 to 17.0)
	Changes after 12 weeks	–0.4 (–6.3 to 5.5)	–2.2 (–8.1 to 3.8)	0.09 (–18.0 to 17.8)
**High-density lipoprotein-cholesterol (mg/dL**)				
	Baseline	42.4 (10.2)	46.3 (12.1)	N/A
	Changes after 6 weeks	–0.9 (–2.7 to 0.9)	–1.0 (–2.7 to 0.8)	–0.9 (–3.9 to 2.1)
	Changes after 12 weeks	2.0 (0.1 to 4.0)^c^	–1.5 (–3.4 to 0.5)	1.1 (–2.6 to 4.9)
**Low-density lipoprotein-cholesterol (mg/dL**)				
	Baseline	112.5 (38.3)	112.5 (38.3)	N/A
	Changes after 6 weeks	1.3 (–5.4 to 8.1)	2.9 (–3.8 to 9.6)	0.2 (–13.7 to 14.0)
	Changes after 12 weeks	1.0 (–5.3 to 7.3)	–1.7 (–4.7 to 8.2)	–5.6 (–25.8 to 14.5)
**Body weight (kg)**				
	Baseline	89.1 (21.8)	90.4 (21.8)	N/A
	Changes after 6 weeks	–0.2 (–0.8 to 0.5)	0.1 (–0.5 to 0.7)	–0.9 (–3.5 to 1.8)
	Changes after 12 weeks	–0.8 (–1.6 to –0.0)^c^	–0.1 (–0.8 to 0.6)	–0.5 (–4.1 to 3.0)
**BMI (kg/m^2^)**				
	Baseline	32.2 (6.3)	32.5 (6.4)	N/A
	Changes after 6 weeks	–0.1 (–0.3 to 0.1)	–0.0 (–0.2 to 0.2)	0.3 (–1.3 to 0.6)
	Changes after 12 weeks	–0.3 (–0.5 to –0.0)^c^	0.0 (–0.3 to 0.2)	–0.4 (–1.5 to 0.6)
**Waist circumference (cm)**				
	Baseline	107.7 (14.5)	108.3 (14.5)	N/A
	Changes after 6 weeks	–0.5 (–1.4 to 0.3)	0.4 (–0.4 to 1.3)	–0.9 (–2.5 to 0.7)
	Changes after 12 weeks	–1.0 (–2.1 to 0.0)^c^	0.6 (–0.4 to 1.6)	–1.1 (–3.1 to –0.8)^d^
**Hip circumference** **(cm)**				
	Baseline	111.2 (14.1)	113 (15.3)	N/A
	Changes after 6 weeks	–0.7 (–1.6 to 0.3)	0.1 (–0.8 to 1.0)	0.0 (–2.0 to 2.0)
	Changes after 12 weeks	–1.0 (–2.0 to –0.0)^c^	0.0 (–0.9 to 1.0)	–2.0 (–4.1 to 0.3)
**Waist-to-hip ratio**				
	Baseline	1.0 (0.1)	1.0 (0.1)	N/A
	Changes after 6 weeks	0.00 (–0.01 to 0.01)	0.00 (–0.01 to 0.01)	–0.04 (–0.01 to 0.1)
	Changes after 12 weeks	–0.0 (–0.01 to 0.01)	0.00 (–0.01 to 0.02)	–0.01 (–0.08 to 0.06)

^a^Values are expressed as mean (SDs) or  mean differences (95% CI).  Comparisons between groups with 1-way analysis of variance and comparisons between groups after 6 and 12 weeks of intervention with analysis of covariance adjusted for baseline levels of each variable, age, sex, use of hypoglycemic treatment (yes/no), and BMI are presented.

^b^N/A: not applicable.

^c^*P*<.05 between before and after the intervention.

^d^*P*<.05 between-group changes.

^e^*P*<.001 between before and after the intervention.

^f^BMI was classified according to the World Health Organization categories: normal weight: <25 kg/m^2^; overweight: 25 to <30 kg/m^2^; obesity class 1: 30 to <35 kg/m^2^; obesity class 2: 35 to <40 kg/m^2^; and obesity class 3: ≥40 kg/m^2^.

### Dietary Intake of Key Foods and Nutrients

As shown in Table S2 in Multimedia Appendix 1, after 12 weeks of intervention, the Greenhabit group exhibited significant increases in the consumption of virgin olive oil (*P*<.001), nuts (*P=*.008), and legumes (*P=*.04). By contrast, the control group experienced a significant reduction in vegetable intake (*P=*.02), along with a significant increase in the consumption of refined cereals (*P=*.049), red wine (*P*=.001), and pastries, cakes, or sweets (*P=*.02). The 14-item Mediterranean Diet adherence score improved by 0.4 points in the intervention group after 12 weeks; however, this increase did not reach statistical significance (*P=*.16).

Regarding nutrient intake (Table S3 in Multimedia Appendix 1), the intervention group showed a significant increase in the consumption of total fat (*P=*.049) and monounsaturated fatty acids (*P=*.009). Between-group comparisons confirmed that the intervention group had higher consumption of total fat and monounsaturated fatty acids compared with the control group (*P*=.02 and *P*=.01, respectively).

### Adherence to the Greenhabit Intervention

The data from the app revealed that users spent an average of 8 minutes per day online, with participants achieving an average of 372 challenges over the 12-week period. Based on the app data for each Greenhabit participant, dietitians offered personalized nutritional and lifestyle advice to address areas where participants scored lower.

As shown in Table 3 and Table S4 in Multimedia Appendix 1, the control group experienced a significant reduction in perceived social support (*P*<.001), along with a significant increase in anxiety (*P=*.03) and body pain perception (*P=*.049). By contrast, the intervention group demonstrated an improvement in social functioning.

**Table 3:** Changes in health-related quality of life at baseline and after 12 weeks of follow-up in the 2 study groups of the Greenhabit randomized controlled trial^a^.

Variables		Greenhabit (n=50)	Control (n=53)	Differences between groups (Greenhabit vs control)
**DUKE—perceived social support (points)**				
	Baseline	53.5 (22.6)	59.9 (24.3)	N/A^b^
	Changes after 12 weeks	–5.3 (–11.8 to 1.3)	–15.2 (–22.2 to –8.1)^c^	7.56 (–3.94 to 19.06)
**The Hospital Anxiety and Depression Scale—anxiety (points)**				
	Baseline	17.2 (1.8)	17.3 (2)	N/A
	Changes after 12 weeks	0.2 (–0.2 to 0.6)	0.5 (0 to 1)^c^	–0.36 (–0.98 to 0.27)
**36-Item Short Form—vitality**				
	Baseline	53 (16.8)	59.9 (16.2)	N/A
	Changes after 12 weeks	0.5 (–2 to 2.9)^c^	0.3 (–2.1 to 2.8)	0.12 (–3.36 to 3.59)
**36-Item Short Form—social functioning**				
	Baseline	74.4 (28.2)	86.0 (18.1)	N/A
	Changes after 12 weeks	5.2 (1.5 to 9)^c^	–2.9 (–6.7 to 0.8)	8.14 (2.84 to 13.44)
**36-Item Short Form—body pain**				
	Baseline	55.7 (34.8)	66.3 (24.8)	N/A
	Changes after 12 weeks	0.8 (–3.7 to 5.3)	4.5 (0 to 9.1)^c^	–3.72 (–10.12 to 2.68)
**Physical activity (** **metabolic equivalent of task** **minutes/week)**				
	Baseline	2020 (2390)	2370 (3995)	N/A
	Changes after 12 weeks	540.7 (99.6 to 981.9)^c^	479.7 (0 to 959.3)^c^	61.08 (–590.6 to 712.75)
**Walking (** **metabolic equivalent of task** **minutes/week)**				
	Baseline	1415 (1285)	1512 (1916)	N/A
	Changes after 12 weeks	441.3 (54.2 to 828.3)^c^	370.6 (–44.8 to 785.9)	70.71 (–497.01 to 638.44)

^a^Values are expressed as mean (SDs) or  mean differences (95% CI).  Comparisons between groups with 1-way analysis of variance and comparisons between groups after 6 and 12 weeks of intervention with analysis of covariance adjusted for baseline levels of each variable are presented.

^b^N/A: not applicable.

^c^*P*<.05 between before and after the intervention.

Finally, physical activity significantly increased in both groups, but only the International Physical Activity Questionnaire test revealed significant changes in MET-walked minutes per day for participants in the intervention group (*P=*.048).

Data from the app are presented in Figure S1 in Multimedia Appendix 1. After 12 weeks, all participants in the Greenhabit group showed improvements in their quality of life: increase in energy (10/76, 13%), happiness (11/85, 13%), positivity (14/78, 18%), social engagement (7/81, 9%), and work-life balance (6/62, 10%).

### Participant Retention

Notably, of the 61 participants, 46 (75%) used the app, with 38 using it daily and participating in the challenges offered. Among the 46 active participants, 15 exhibited intermittent use of the app. The data provided by the app revealed that the average daily time spent online was 8 minutes, and the average number of challenges completed during the 12 weeks was 372.

## Discussion

### Principal Findings

The primary goal of our RCT was to evaluate the effectiveness of the Greenhabit method in promoting self-management and self-care among patients aged 18-75 years recently diagnosed with T2D. Significant improvements in HbA_1c_ and FPG concentrations were observed in both the intervention and control groups at 6 and 12 weeks. However, notable between-group differences were evident in secondary outcomes, particularly in WC after 12 weeks. Additionally, the Greenhabit group showed significant improvements in BP, triglycerides, HDL-cholesterol, BW, HC, and overall quality of life following the 12-week intervention period.

Notably, the Greenhabit app proved more effective in significantly reducing anxiety, fostering positive emotions, improving coping with body pain, and enhancing participants’ perception of health. By contrast, the control group showed either no improvement or worsening trends in these areas.

### Changes in T2D Parameters

Results indicate that telemedicine, particularly through the Greenhabit app, effectively reduced FPG and HbA_1c_ levels within 6 weeks of intervention, with these effects sustained at 12 weeks. However, no significant differences were found between the intervention and control groups. The app’s visual representation of blood glucose values may have contributed to participant motivation.

Comparison with other studies showed similar HbA_1c_ reductions in the intervention group, but no significant differences between groups [46,47], suggesting that the app alone may not drive sufficient lifestyle changes, at least in the short term. The effectiveness may be enhanced through health care provider support and a personalized follow-up plan, which many patients may require to achieve their goals [48]. Several factors may explain the lack of differences in HbA_1c_ levels between groups as the primary outcome. The Hawthorne effect, where awareness of being observed influences behavior, may have led to improved self-management in both groups [49]. Additionally, baseline HbA_1c_ levels below 8% and the potential for increased attention from general practitioners during the RCT could have influenced the outcomes [50,51].

In patients with T2D, diet and exercise play a vital role in glycemic control and the prevention of cardiovascular risk [26,52-55]. However, conventional education may be insufficient to improve self-care, as patients with T2D often resist changing unhealthy behaviors [56]. This point will be explored further later.

It is important to highlight that the high costs associated with T2D are often due to the condition being accompanied by additional health-related complications. Moreover, health-related quality of life has been found to be significantly lower in individuals with T2D-related complications compared with those without such complications [57]. A 1% reduction in HbA_1c_ has been reported to significantly decrease microvascular and macrovascular complications in patients with T2D [58]. In our study, the intervention group showed a reduction in HbA_1c_ below 7%, which is associated with maximum cardiovascular benefits [59]. After 12 weeks, we hope participants will feel more aware, resilient, and energized, enabling them to maintain these habits long-term, though this would require further evaluation.

### Changes in Cardiovascular Risk Factors

Remarkably, the mHealth intervention with Greenhabit led to improvements in cardiometabolic parameters, including systolic and diastolic BP, triglycerides, and HDL cholesterol, as well as adiposity indicators such as BMI, WC, and HC after the 12-week intervention. These findings are consistent with those of Hamaya et al [60], who reported significant reductions in BW and HbA_1c_ and an increase in HDL-C among 12,602 healthy participants (mean age 44.1 years, SD 10.2 years) using the Kencom mHealth app. A Chinese meta-analysis [61] and a recent systematic review [62] indicated that mHealth app interventions were associated with significant improvements in metabolic measures (HbA_1c_, BW, BP, lipid profile, and physical activity) in patients with T2D. This suggests that mHealth apps, even when used by older individuals, can provide valuable support to enhance diabetes self-care and thereby improve clinical outcomes.

### Cognitive and Behavioral Improvements

Participants in the intervention group reported a more positive outlook, improved coping with body pain, and favorable changes in their perception of health. These positive outcomes are attributed to the social support provided by the app environment, including the role of supportive peers and the broader social network. While no changes in social aspects were observed in the intervention group, the control group showed a significant decrease. Notably, there were differences between the groups in terms of anxiety, with a tendency to decrease in the intervention group and an increase in the control group. This aligns with previous findings suggesting that implementing support mechanisms can reduce anxiety [63,64].

The primary objective of this mHealth app targeting anxiety or depression is centered around cognitive-behavioral education. The Greenhabit method is based on educating individuals to cultivate enduring habits. By adopting a holistic approach that encompasses physical, mental, and social dimensions, it fosters resilience and self-awareness, thereby promoting the adoption of healthy habits. Moreover, fostering social engagement empowers individuals to take control of their health. Human interactions, particularly with medical physicians or health care professionals, along with the provision of social support and an appealing mHealth app interface, play pivotal roles in enhancing compliance, adherence, dropout rates, and treatment efficacy [12]. First, the combination of automatic information through the Greenhabit app, face-to-face visits with a dietitian, and regular phone calls during the 12-week intervention likely contributed to maintaining healthy behaviors such as diet. Furthermore, the critical support provided by nurses proved invaluable when participants encountered personal difficulties. The nurse served as a supporter, helping to motivate the patients. Interactions also created accountability, focus, and awareness of how behaviors impacted the participants’ health. Thus, the combination of nursing assistance and the mHealth app demonstrated short-term effectiveness in enhancing diabetes self-efficacy and significantly increasing physical activity within the intervention group. These support mechanisms may explain the observed reductions in FPG and HbA_1c_ levels, as well as improvements in anxiety and general health status. The interaction with health care providers and the use of devices seem to enhance feelings of support for some individuals [65]. Second, while the influence of social support and its resultant outcomes may resemble those observed in conventional standards of care, a distinguishing characteristic of the digital social support offered by this mHealth app is the provision of anonymity and accessibility. The capability to exchange comparable lived experiences of anxiety or depression among peers, coupled with being accountable to an authoritative figure (eg, health care professional) for symptom monitoring or assessment, holds the potential to bolster engagement and adherence to the mHealth app. Finally, the Greenhabit app demonstrates attributes of easy navigation, relatability, engagement, and aesthetic appeal, ensuring greater user engagement.

### Adherence to the Intervention

The observation that not all participants consistently used the app (46/61) concurs with findings from Torbjørnsen et al [66], indicating varying acceptability of T2D self-management apps based on factors such as sex, age, education, expectations, and ease of use. Some participants viewed the app as more of a time-waster than a helpful tool, citing forgetfulness or excessive time requirements. Despite this, most participants expressed satisfaction (43/49, 88%) and considered the app useful (42/49, 86%), noting that it established new routines resulting in an improved lifestyle.

### Influence of the mHealth App on Diabetes Self-Management and Treatment

The level of self-management may vary between individuals with T2D and those with type 1 diabetes, potentially influenced by treatment intensity and the necessity for regular blood glucose monitoring, particularly among insulin-dependent individuals [67]. To underline, most of our participants indicated that they were already self-monitoring their blood glucose levels at the outset of the study, implying a preexisting engagement in self-management practices regardless of insulin use.

To achieve a meaningful improvement in HbA_1c_ levels and cardiovascular risk factors in participants with T2D through self-management interventions, it is essential to first enhance healthy eating habits, promote engagement in physical activity, and ensure consistent adherence to medication. It is well-known that patients with T2D usually show higher resistance to changing their unhealthy behaviors, particularly when it comes to increasing physical activity and improving dietary habits [13,26]. It has been demonstrated that physical activity may contribute to a 30%-50% reduction in the development of T2D [68]. While both groups showed similar changes in T2D management strategies, including diet and physical activity, only the intervention group demonstrated a significant improvement in exercise. The control group did not show significant improvement in overall diet and displayed reduced adherence to the Mediterranean Diet. These findings are consistent with those of other studies [53-55].

Neither significant differences in dietary habits were noted between the intervention and control groups nor were significant changes observed in dietary habits after 12 weeks. This may be attributed to limited app usage, consistent with findings by Agarwal et al [11], and the inherent challenges in altering dietary habits influenced by external (food industry) and internal (long-term preferences) factors [69]. Additionally, participants using the Greenhabit app had to manually input data, which posed a barrier for some, leading to dropouts, including those aged 60 years and above and younger individuals who never used the app. A Norwegian RCT reported preferential app usage by older compared with younger participants [12].

However, the acquisition and maintenance of a healthy lifestyle, based on a healthy dietary pattern and physical activity programs, could be insufficient. As the intrinsic motivation of participants plays a key role, it may be recommended to include a motivational approach to bring about behavioral change [56]. Participants allocated to the Greenhabit group are more aware of what they eat and the daily exercise they perform, which may explain better control of the disease.

Chronic diseases carry severe consequences, including reduced quality of life, health emergencies, complications, and death [70]. Managing them poses significant challenges and costs for health care systems globally, with chronic diseases accounting for 71% of all deaths annually [71]. These diseases exacerbate inequality, disproportionately affecting socially disadvantaged individuals and hindering poverty reduction efforts [30]. Improving patients’ health not only enhances their quality of life but also alleviates societal economic burdens. Effective management relies on understanding the impact of chronic diseases on patients’ daily lives and empowering them to self-manage and make informed decisions [14]. This approach aligns with national health care strategies emphasizing personalized medicine and self-management [72]. Patient empowerment involves actively engaging in health responsibilities and promoting meaningful involvement in treatment decisions [70]. In this regard, mHealth emerges as a cost-efficient tool to support self-management and foster empowerment.

### Limitations and Strengths

The study’s primary strength lies in its design as an RCT, which allows for causal inference. Other strengths include a sizable and diverse sample (ages 18-75 years, gender-balanced), standardized data collection conditions for anthropometric and blood measurements, and the option for participants to involve a mate in app usage for mutual support. The app’s holistic approach—addressing diet, physical activity, positive thinking, relaxation, and social aspects—distinguishes it from other mHealth strategies. This comprehensive strategy potentially explains its success in reducing cardiovascular risk factors. Our study stands as one of the few RCTs that specifically investigated the impact of using a mobile phone app as a digital diabetes diary to enhance quality of life.

Our study also has limitations. It was short-term (12 weeks) and conducted in a single center, which means findings may not be generalizable to non-T2D populations and could be subject to selection bias among patients with T2D. Additionally, technological challenges, particularly for older individuals, could hinder the app’s use as a public health strategy. Furthermore, individuals from low-resource areas may struggle to access smartphone technology due to associated costs, in both acquiring the device and downloading paid apps. Additionally, selection bias occurred because participants were required to possess a smartphone. These financial barriers can limit access to mHealth interventions, exacerbating inequalities in health care access. The findings might not be applicable to cohorts with lower levels of motivation or limited technological knowledge. The observed increase in skill and technique acquisition may imply a heightened capability among participants to alleviate symptoms related to T2D and effectively manage their overall health. This enhancement may encompass improved utilization of technical aids. Although our study did not incorporate a specific health education impact questionnaire, the integration of validated health-related quality-of-life assessments could provide valuable insights into the observed improvements in self-management abilities. Finally, while HbA_1c_ is crucial for long-term glycemic control, the exclusion of glycemic variability assessment limits a comprehensive understanding of glycemic status. Combining HbA_1c_ and glycemic variability could enhance diabetes management strategies [3]. More RCTs with longer follow-up times should be conducted to evaluate the long-term effect of diabetes-related mobile apps on glucose control and quality of life and to confirm that the outcomes seen in initial studies are sustainable over time.

### Conclusions

Chronic illnesses can have significant consequences, often necessitating patients to depend on lifestyle choices and effective coping strategies for management. Self-management, which empowers patients to take control of their own health, is crucial. Studies on self-management highlight the importance of patient empowerment, enhancing their autonomy in health-related decisions. mHealth offers an economical means to enhance self-management and empower individuals. The integration of the Greenhabit app into clinical practice has the potential to positively impact glycemic control, reduce anxiety, and improve overall health status in individuals with T2D. Therefore, the Greenhabit app could be an additional supportive tool in the comprehensive management of patients with T2D within clinical settings.

The use of tailor-made interventions supported by an artificial intelligence–based infrastructure to enhance health and achieve sustainable behavior change represents a significant and promising approach for T2D self-management. However, larger studies are required to validate the effectiveness of these interventions.

## Appendix

Multimedia Appendix 1 Additional analyses and statistics.

Multimedia Appendix 2 CONSORT (Consolidated Standards of Reporting Trials)‐EHEALTH checklist (V.1.6.1).

## Abbreviations

ANCOVA: analysis of covariance
ANOVA: analysis of variance
BP: blood pressure
BW: body weight
FPG: fasting plasma glucose
HbA1c: glycated hemoglobin
HC: hip circumference
HDL: high-density lipoprotein
IPAQ: International Physical Activity Questionnaire
mHealth: mobile health
RCT: randomized controlled trial
T2D: type 2 diabetes
WC: waist circumference

## Data Availability

Data availability for the Greenhabit trial is restricted due to signed consent agreements, which permit data sharing only with external researchers conducting studies aligned with the project’s aims. Researchers interested in accessing the Greenhabit trial data used in this study may contact the corresponding author.
