# Supplementary material for: Efficacy of a Mobile Health–Based Behavioral Treatment for Lifestyle Modification in Type 2 Diabetes Self-Management: Greenhabit Randomized Controlled Trial
Source: J Med Internet Res. 2025 Jan 22;27:e58319. doi: 10.2196/58319 (PMC11799821; doi:10.2196/58319)
Supplement: Multimedia Appendix 1 [file jmir_v27i1e58319_app1.docx]

**Appendix**

**Supplementary methods**

Greenhabit Behavioral Treatment program

*Aim:*

The Greenhabit app emphasizes personal growth, covering areas such as proactivity, handling work pressure, managing situations that disrupt balance, building self-confidence, and fostering resilience. With a weekly thematic focus, the app guides users through a personal growth program, encouraging reflection on individual situations. This approach helps users take control of their lives and make choices that promote personal well-being (<https://greenhabit.nl/en/what-is-greenhabit/> [23]).

*Cognitive Behavioral Therapy, AI & Gamification:*

The Greenhabit program (mHealth) aimed to steer a sustainable adoption of a healthy lifestyle by increasing resilience and promoting healthy habits using gamifications strategies with interventions grounded in psychological theories. The integrated buddy system and community with peers contributes to social integration and motivation. The online coach provides support via the connected dashboard when needed (https://greenhabit.nl/en/).

*Holistic approach (physical, social, and emotional):*

Greenhabit is a holistic self-management training app that provides solutions (grounded in psychological theories) for durable behavioral changes towards a healthier lifestyle. It also involves the environment, which has a major influence on patient behavior (<https://greenhabit.nl/en/> [29]). This behavioral change game is an intelligent life journey in which, every day, for 12 weeks, users must open a treasure chest containing advice, encouraging phrases, challenges, and self-monitoring [BW, fasting glucose, BP, physical activity (step counter), medication use, food intake, health status and quality of life]. The 5 pillars on which the game is based are: i) healthy diet (the advice given refers to the Mediterranean diet and suggests the 80-20 rule: 80% of the food has to be healthy while the other 20% could be less healthy); ii) exercise (approximately 30 minutes per day); iii) positive thinking (reflecting on positive things that happen in the patient’s day to day life); iv) relaxation (at least a moment of relaxation per day); and v) social environment (at least one social gathering a day). Thus, Greenhabit mixes physical, social, and emotional, 360-degree approaches for better management of a chronic disease that requires significant life changes. The impact of Greenhabit on the cardiovascular burden of disease is an improvement of quality of life due to improvement of lifestyle behavior and a reduction of cardiac re-events (<https://greenhabit.nl/en/> [29]).

The development of Greenhabit behavioral program was based on these methods:

– Brain learning techniques (1)

– The Behavior Change Wheel for behavioral change (2)

– Behavioral Change Techniques (3)

-Artificial Intelligence -development of new habits (4)

*New Healthy Habits:*

This method is an educational approach based on learning new sustainable habits; the brain needs between 28 to 84 days to form a habit, depending on time spent per day (5). To date, a study published in the European Journal of Social Psychology found that, on average, 66 days were required to develop a habit, with a range between 18 and 254 days (4). Aligning with the findings observed by Lally et al., a study published in the British Journal of Health Psychology in 2021 found that people need an average of 59 days to successfully form a new habit in nutrition (6).

*Tackling the causes of Type-2 Diabetes and Obesity:*

Greenhabit, the cure for chronic diseases and healthy aging, has developed a T2D treatment program together with Grendel Games, University Barcelona, and Hospital Clinic Barcelona. A Holistic gamified behavioral Health treatment aimed at tackling the causes of T2D and obesity. Greenhabit is able to personalize the treatment based on the underlying causes behind the disease. The app has been designed specifically for patients with T2D. However, the in-app experience is subjective to each participant. In fact, at the beginning of the "game", the subject is asked about what goals he or she wants to achieve (eat healthier, lose weight, become more active, be more relaxed, etc.) and depending on what is chosen, the app will offer a distinct path to support this.

*Greenhabit box & reward mechanism:*

Through completing the task and sharing the achievements with the community (by posting pictures or post with thoughts), the user earns points. When enough points are earned, the user can open a physical reward: with the registration, the participant receives a Greenhabit box containing different rewards (jumping rope, book with recipes, etc.) to be opened when the patient has earned enough points. Motivation, training, and reward mechanisms play a crucial role in the Greenhabit Health behavioral change processes.

The patient has the possibility to participate in this game with a buddy; someone from the private environment, chosen by the patient himself. The patient can also decide whether to expand his community by connecting with other participants/sufferers through the Greenhabit community in the App, thus creating a social support circle.

The idea is that through completing challenges, rewarding healthy behaviors, having a friend (buddy) participating at the same time, and being in a community of people with the same disease and goals, there is better engagement and change toward healthier behaviors (7).

Greenhabit Method

We believe that the future of self-management healthcare is a world in where prevention and healthy life expectancy are key. The only way to be at your best is to start from a healthy lifestyle. Greenhabit guides the creation of habits that deliver a positive impact to your life.


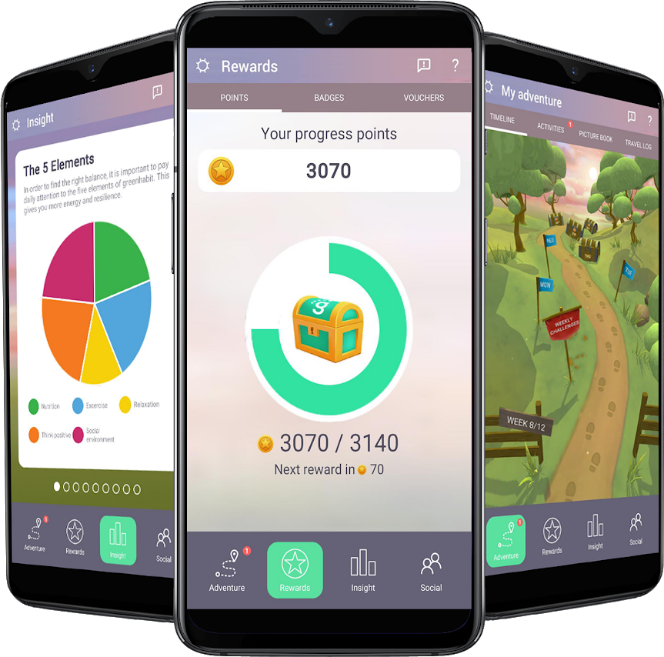

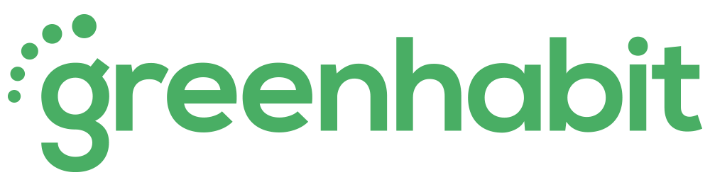


*How to build sustainable healthy habits in twelve weeks*

*Introduction*

Worldwide, diseases are often treated symptomatically. For instance, doctors in the Netherlands have received only 40 hours of education in lifestyle improvement (healthy food, exercise), during their 6-year training . A doctor is only called when it is “too late” and there are already complaints related to a clinical picture. The result is most often a curative treatment for the patient instead of a preventive treatment or the combination of those two.

As a result the common belief is that health is a matter for the doctor. If you don't feel well, go to the doctor and he will fix it. Over the past years the awareness has arisen that the population must be educated on chronical disease prevention and lifestyle improvement to prevent and reduce chronical diseases.


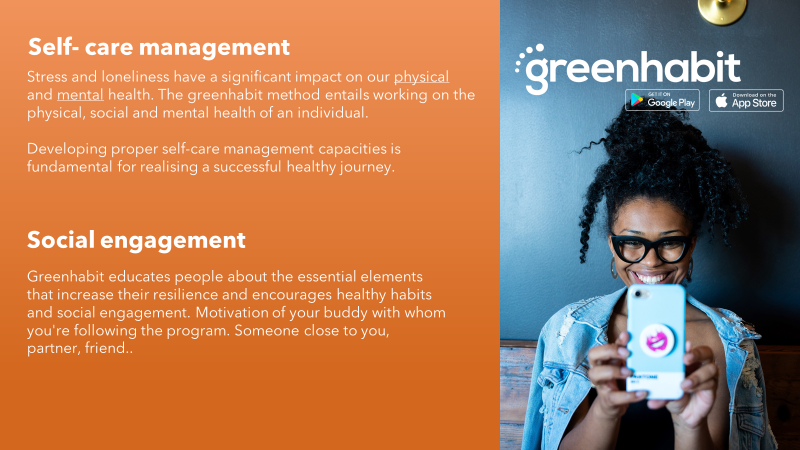


Based on these insights greenhabit has developed a method to educate and support people with sustainable healthy habits and resilience. The greenhabit method is based on two theories:

1. The behavioral change Wheel. We have used this theory to create the interventions and techniques within greenhabit.
2. McClelland Iceberg model. We have used this model as a framework for building the content and challenges and measures for the users of greenhabit.

Both models were used for the development of the greenhabit education. As a result greenhabit is an App (mHealth) for Android and IOS devices with gamification and personal development.

*Greenhabit*

Greenhabit is an holistic education that put people in control of their health by developing sustainable behaviour change. The holistic approach (physical, mental and social) provides mental resilience and self-awareness. The social integration (buddy and community) provides motivation and social attention to maintain healthy habits. It generates motivation to take care of your health by stimulating with nudges to nutrition, physical activities and social interactions to maintain your wellbeing. The game/app uses technology of serious gaming. The vitality education is a journey of twelve weeks in where the player works on the 5 elements of greenhabit. (nutrition, exercise, relaxation, Positive mindset and social environment). We use the power off brain learning, like nudges, content, learnings, challenges, rewards, working with a buddy, using the senses and joy. The brain is plastic and we can learn new habits. It takes at least 68 days to learn new habits. After twelve weeks, players feel more aware, they have more resilience and energy.


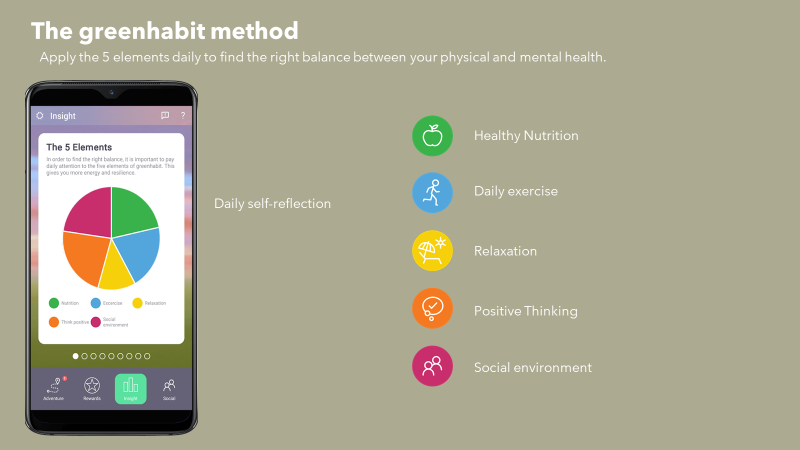


*How is greenhabit constructed*

The greenhabit method is a combination of online and offline interventions. The philosophy is summarized in a book which the player receives in a box at home or at work together with a water bottle, a small journal and some items to stimulate healthy habits. The online education is offered in an App with gamification. For the development of this App the Behavioral change wheel is used.


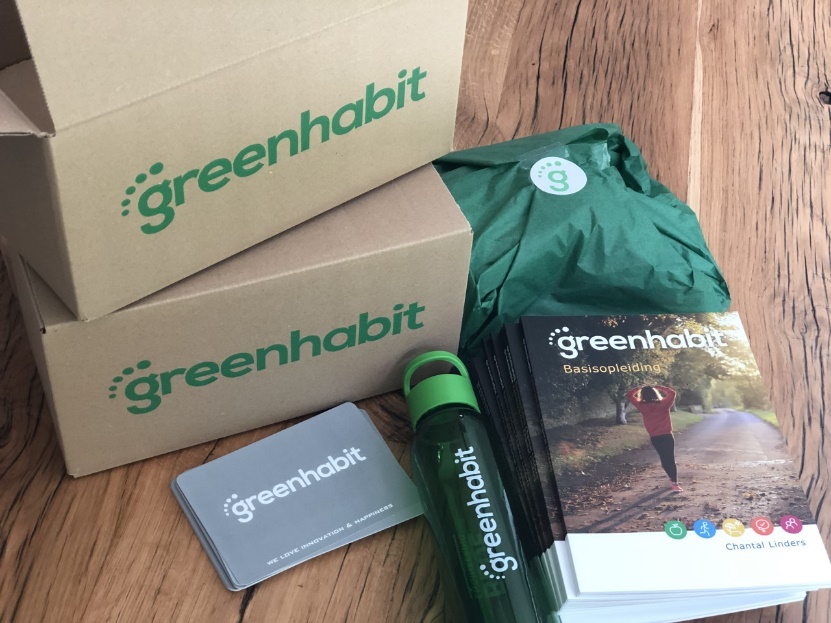


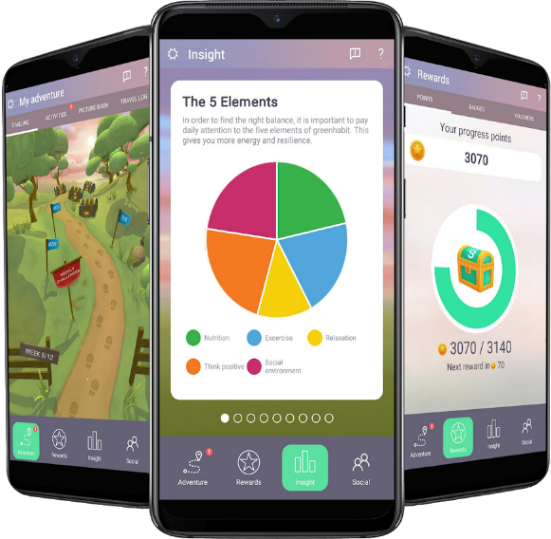


*Behavioral Change Wheel*


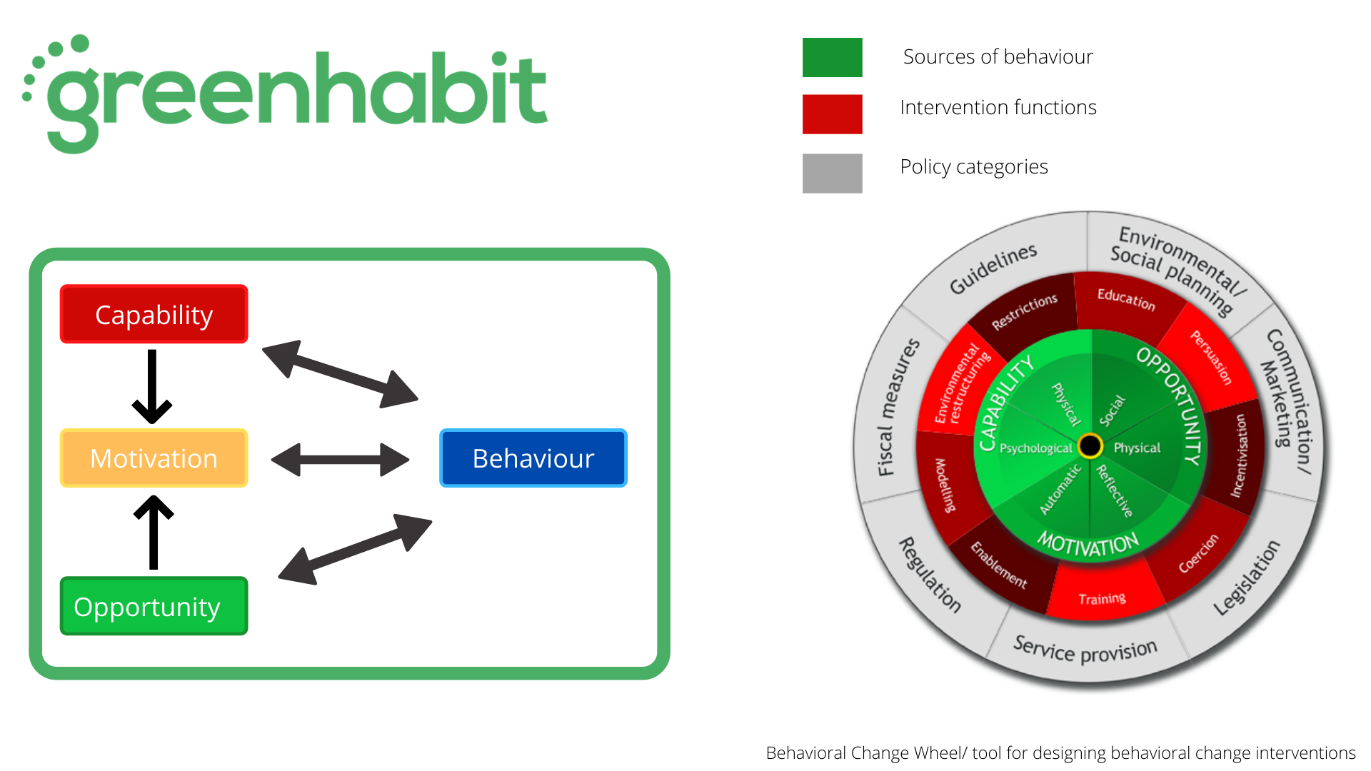


*Intervention functions*In the greenhabit game the player is influenced to optimize the sources of behavior. To create behavior change the three sources must be aligned for this purpose. If one of the three sources is not sufficient available, the attempt for behavioral change will not be successful. To start with greenhabit you need an intrinsic motivation and awareness that change is necessary.

The following intervention functions are deployed in the game to influence the source:

| **Source** | **Intervention function** | **Greenhabit intervention technique** |
| --- | --- | --- |
| *Capability*  *Motivation*  *Opportunity* | *Education*  Increasing knowledge or understanding. For instance: Providing information to promote healthy eating | 1. *E-learning* – challenge cycle and measure system to realize awareness |
|  | *Training*  Imparting skills  For instance: Advanced driver training to increase safe driving | 1. *Challenges* to practice on the five elements |
|  | *Modelling*  Providing an example for people to aspire to or imitate.  For instance: Using TV drama scenes involving cycling to the office or school to increase the use of a bike for travelling to work or school. | 1. *Pictures* in the social (tab) of recipes, challenges of buddies and friends as an inspiration for healthy food and exercise. |
|  | *Persuasion*  Using communication to induce positive or negative feelings or stimulate action. For instance: Using imagery to motivate increases in physical activity | 1. *Nudging* and Challenges in combination with attractive images of e.g. nutrition or exercise to create an activating effect. |
|  | *Coercion*  Creating expectation of punishment or cost.  For instance: Raising the financial cost to reduce excessive alcohol consumption | 1. *Within greenhabit we do not use Coercion* in a negative way but show the better choices based on explanation about the unhealthy choices |
|  | *Incentivization*  Creating expectation of reward.  For instance: Using prize draws to induce attempts to stop smoking | 1. *Gamification* with game-points and rewards |
|  | *Environmental Restructuring*  Changing the physical or social context. For instance: Agree with your family, to buy no longer soft drinks so it is not available and the switch to drinking water is easier. | 1. *Buddy system* to involve the personal environment and including friends (co-workers) in the “social” of the game. |
|  | *Enablement*  Increasing means/reducing barriers to increase capability or opportunity.  For instance: Behavioural support for smoking cessation, medication for cognitive deficits, surgery to reduce obesity, prostheses to promote physical activity | 1. *With the Buddy* system we stimulate the player to exercise together. With team challenges we influence the co-workers to do a healthy task and this creates a situation that in the office the coworkers talk about the challenges. |
|  | *Restriction*  Using rules to reduce the opportunity to engage in the target behaviour (or to increase the target behaviour by reducing the opportunity to engage in competing behaviours)  For instance: Prohibiting sales of solvents to people under 18 to reduce use for intoxication | 1. A) We use the 20-80% rule in the manner that daily you eat 20% unhealthy or less and at least 80% healthy food.   B) We ask the player to judge their food with a number. If they valuate the food or snack with a number lower than an 8, we advise not to eat or drink it. But if it is an 8 or higher, you can enjoy it but limited of course.  C) Sugar intake max. 60gr |

The Behavioral change interventions inside Greenhabit summarized:


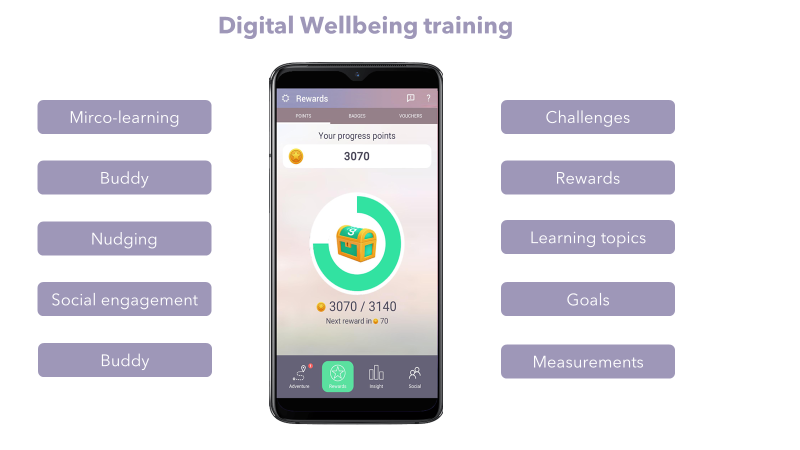
Capability:

Micro Learning

E-learning cycle

Travel log

Measurements/statistics

Motivation:

Buddy system

Nudging

Week goals

Gamification/ rewards

Opportunity:

Social environment

Buddy system

*E-Learning Program*

In the game the participants of the game receive every week a new E-learning theme. This theme is offered to the player in the following E-learning-challenge cycle:

E-learning – challenge cycle

Learning 🡺Practice🡺 reflexing 🡺 repeat

The E-learning program covers the following weekly subjects:

1. Impact of Sugar
2. Stress management
3. Decision making based on principles
4. Proactive thinking
5. Timemanagement
6. Self-confidence
7. Mindfulness
8. Cognitive behavioral therapy method
9. Quality of Sleep
10. Personal effectiveness
11. Happiness
12. Work-Life balance

*Iceberg model McClelland*

Translation of interventions in green habit to McClelland Iceberg model.


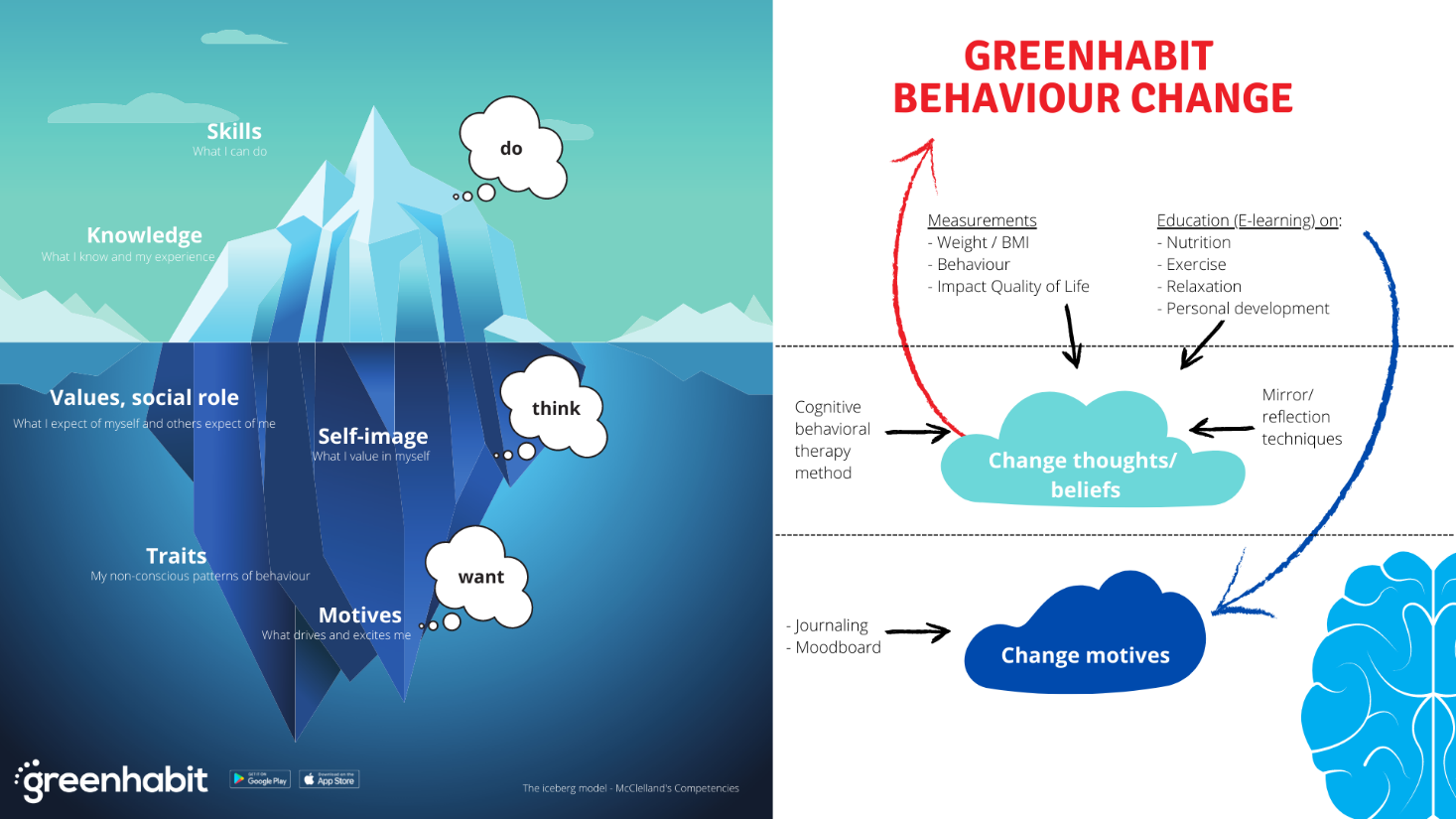


*Brain learning*

Many brain learning techniques have been used in the App greenhabit. People like to learn and prefer to learn together. That's why you always do the education together with a buddy from your private environment and you can find your fellow-sufferers in the community. This social engagement increases the motivation to persevere and gives mental support. Greenhabit increases self-awareness in small steps (mini-goals) and gives small pushes (nudging). The challenges and rewards increase involvement. Every small success gives dopamine in the brain.


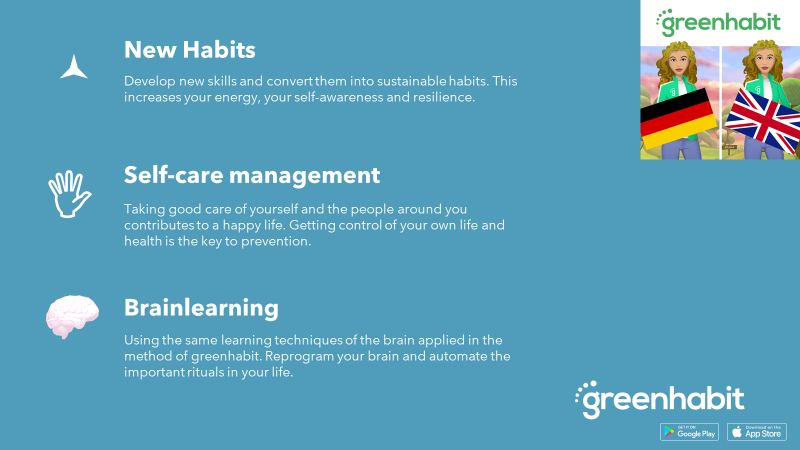


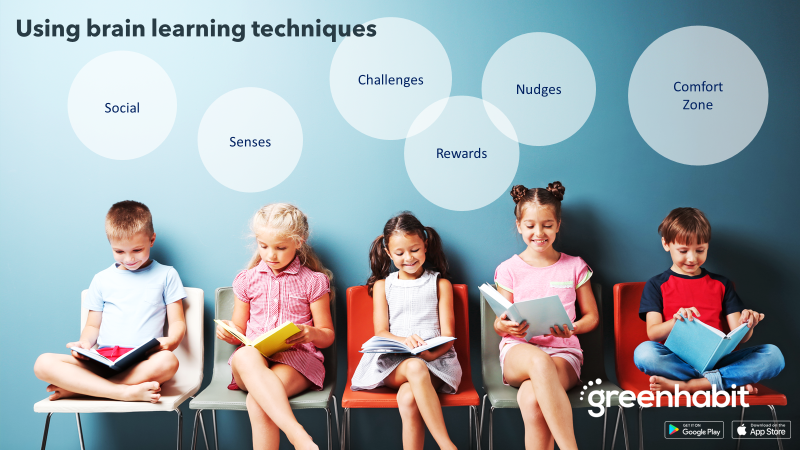


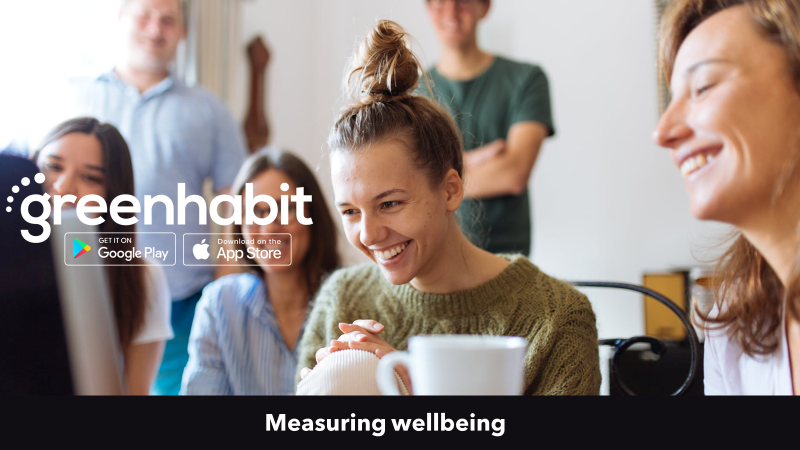


*Dashboard wellbeing*

The wellbeing dashboard (for (Healthcare)organizations) shows the anonymized data at 4 moments


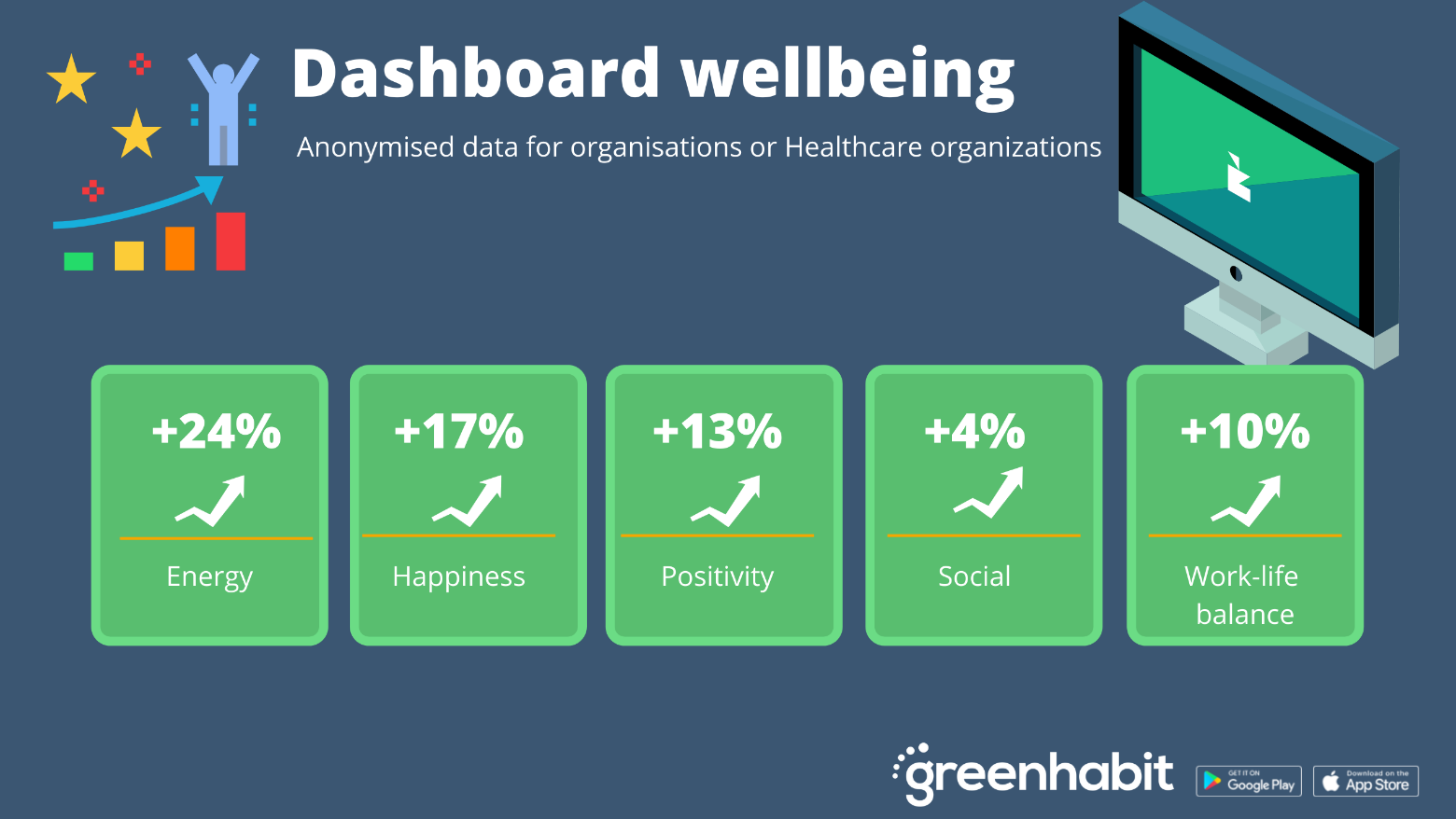


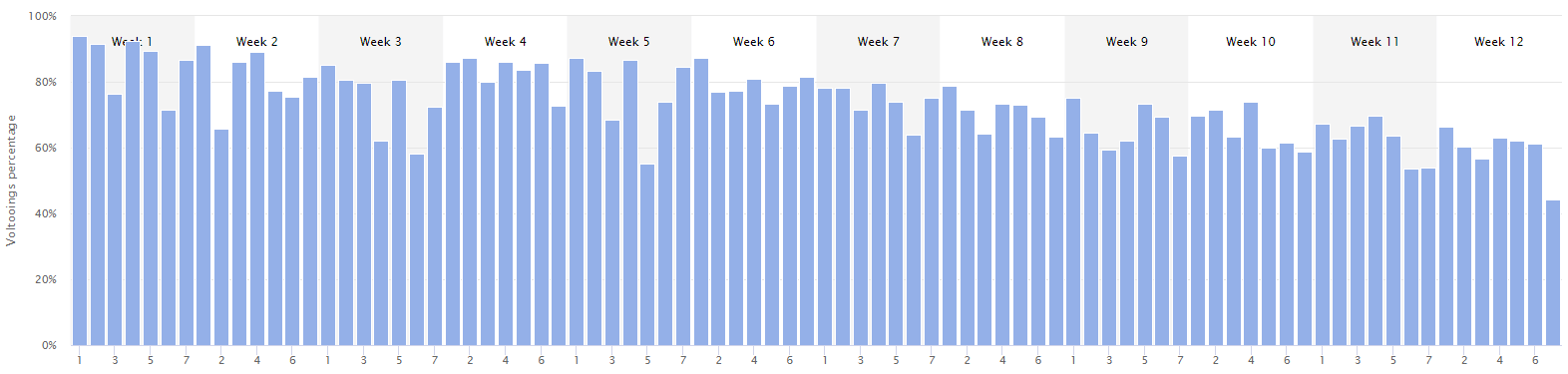
And the dashboard also shows the engagement of the players:

References

1. Greenhabit. What is Greenhabit? Greenhabit. URL: https://greenhabit.nl/en/what-is-greenhabit/[accessed 2024-01-15]
2. [McClelland, D. C.](https://www.toolshero.nl/bekende-auteurs/david-mcclelland/), Koestner, R., & Weinberger, J. (1989). How do self-attributed and implicit motives differ?. Psychological review, 96(4), 690.
3. Michie, S., van Stralen, M.M. & West, R. The behaviour change wheel: A new method for characterising and designing behaviour change interventions. Implementation Sci 6, 42 (2011). https://doi.org/10.1186/1748-5908-6-42
4. Greenhabit. URL: https://greenhabit.nl/en/[accessed 2024-01-15]
5. [McClelland, D. C.](https://www.toolshero.nl/bekende-auteurs/david-mcclelland/) (1987). Human motivation. [CUP Archive](https://www.cambridge.org/).
6. [McClelland, D. C.](https://www.toolshero.nl/bekende-auteurs/david-mcclelland/) (1985). How motives, skills, and values determine what people do. American Psychologist, 40(7), 812.
7. Covey, S. (2010) De zeven eigenschappen van effectief leiderschap.(68e druk). Amsterdam: Business Contact.
8. Dyer. W (1997) Niet morgen maar nu.(45e druk). Utrecht: Bruna uitgevers
9. Wiebes, H. (2016). Het oplichterssyndroom:’de vloek van slimme mensen’. Volkskrant.nl, 23 maart.

**Supplementary Figures and Tables**

**Supplementary Figures**


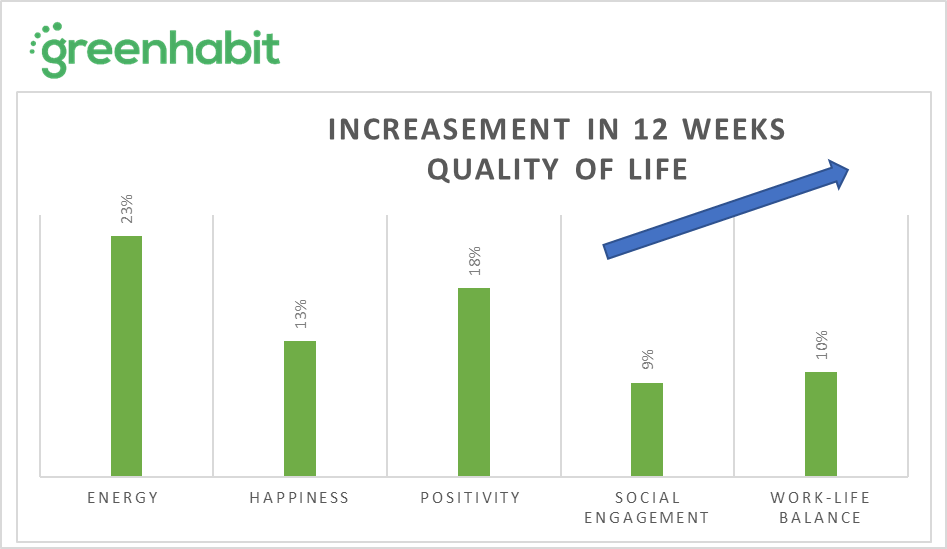
Figure 1. Changes of health-related quality of life after 12- weeks of follow-up in the Greenhabit group. This randomized controlled trial, conducted in Barcelona (Spain), evaluates the efficacy of a mHealth-based behavioral treatment for lifestyle modification in the self-management of type 2 diabetes.

**Supplementary Tables**

Table 1. Changes in blood pressure, cardiovascular risk factors and adiposity, at baseline, and after 6 and 12 weeks of follow-up (unadjusted) in the two study groups of the Greenhabit randomized controlled trial. This trial, conducted in Barcelona (Spain), evaluates the efficacy of a mHealth-based behavioral treatment for lifestyle modification in the self-management of type 2 diabetes.

|  |  | **Greenhabit (n=50)** | **Control (n=53)** |  |
| --- | --- | --- | --- | --- |
|  |  | Mean | Mean | *Differences between groups (Greenhabit vs control)* |
| **SBP, *mmHg*** | Baseline | 141.3±19.5 | 142.5±18.5 |  |
|  | Changes after 6 w. | -3.2 (-7.3, 0.9) | -2.3 (-6.3, 1.7) | -0.9 (-6.6, 4.8) |
|  | Changes after 12 w. | -4.2 (-8.6, 0.2) | -5.3 (-9.5, -1.1)* | 1.1 (-5.0, 7.2) |
| **DBP, *mmHg*** | Baseline | 84.9±10.6 | 81.2±10.1 |  |
|  | Changes after 6 w. | -2.8 (-4.8 to -0.8)* | 0.4 (-1.6, 2.3) | -3.1 (-5.9, -0.3)* |
|  | Changes after 12 w. | -2.3 (-4.3, -0.2)* | -0.5 (-2.5, 1.4) | -1.7 (-4.6, 1.1) |
| **Glucose, *mg/dL*** | Baseline | 125.7±29.1 | 132.4±38.0 |  |
|  | Changes after 6 w. | -6.1 (-10.3, -1.8)* | -5.1 (-9.3, -0.8)* | 0.1 (-6.2, 6.5) |
|  | Changes after 12 w. | -3.5 (-8.6, 1.6) | -1.9 (-6.9, 3.0) | -1.6 (-8.7, 5.5) |
| **HbA1c, %** | Baseline | 6.88±1.12 | 6.90±0.76 |  |
|  | Changes after 6 w. | -0.4 (-0.5, -0.3)** | -0.3 (-0.4, -0.1)** | -0.1 (-0.3, 0.0) |
|  | Changes after 12 w. | -0.4 (-0.6, -0.3)** | -0.3 (-0.4, -0.1)* | -0.1 (-0.4, 0.1) |
| **Triglycerides, *mg/dL*** | Baseline | 157.3±65.0 | 143.1±55.2 |  |
|  | Changes after 6 w. | -11.0 (-24.6, 2.6) | -3.5 (-16.8, 9.9) | 1.9 (-18.4, 22.2) |
|  | Changes after 12 w. | -18.9 (-35.3, -2.7)* | -7.8 (-24.0, 8.4) | 3.1 (-22.2, 28.3) |
| **Total-cholesterol, *mg/dL*** | Baseline | 177.0±40.7 | 181.3±36.3 |  |
|  | Changes after 6 w. | 1.0 (-5.0, 7.1) | 0.6 (-5.3, 6.6) | 0.4 (-8.1, 8.9) |
|  | Changes after 12 w. | 0.4 (-5.5, 6.2) | -2.9 (-8.9, 3.0) | 3.3 (-5.0, 11.6) |
| **HDL-Cholesterol, *mg/dL*** | Baseline | 42.4±10.2 | 46.3±12.1 |  |
|  | Changes after 6 w. | -0.7 (-2.5, 1.0) | -1.1 (-2.8, 0.5) | 0.5 (-1.9, 2.9) |
|  | Changes after 12 w. | 1.8 (-0.2, 3.8) | -1.2 (-3.2, 0.7) | 3.9 (0.6, 7.1)* |
| **LDL-Cholesterol, mg/dL** | Baseline | 112.5±38.3 | 111.2±32.9 |  |
|  | Changes after 6 w. | 2.4 (-4.2, 9.0) | 1.8 (-4.7, 8.4) | 0.6 (-8.7, 9.9) |
|  | Changes after 12 w. | 1.3 (-4.9, 7.5) | 1.4 (-4.9, 7.8) | -0.1 (-9.0, 8.8) |
| **BW, *Kg*** | Baseline | 89.1±21.8 | 90.4±21.9 |  |
|  | Changes after 6 w. | -0.1 (-0.8, 0.5) | 0.0 (-0.6, 0.6) | -0.1 (-1.0, 0.7) |
|  | Changes after 12 w. | -0.8 (-1.5, -0.0)* | -0.1 (-0.8, 0.6) | -0.7 (-1.7, 0.4) |
| **BMI, *kg/m^2^*** | Baseline | 32.2±6.3 | 32.5±6.4 |  |
|  | Changes after 6 w. | -0.06 (-0.3, 0.2) | -0.01 (-0.2, 0.2) | -0.0 (-0.3, 0.2) |
|  | Changes after 12 w. | -0.3 (-0.5, -0.0)* | -0.06 (-0.3, 0.2) | -0.2 (-0.6, 0.1) |
| **WC, *cm*** | Baseline | 107.7±14.5 | 108.3±14.5 |  |
|  | Changes after 6 w. | -0.5 (-1.3, 0.4) | 0.4 (-0.5, 1.2) | -0.0 (-0.3, 0.2) |
|  | Changes after 12 w. | -1.0 (-2.1, 0.0) | 0.6 (-0.4, 1.6) | -1.5 (-3.0, -0.0)* |
| **HC, *cm*** | Baseline | 111.2±14.1 | 113±15.3 |  |
|  | Changes after 6 w. | -0.7 (-1.7, 0.2) | 0.1 (-0.8, 1.0) | -0.8 (-2.1, 0.5) |
|  | Changes after 12 w. | -1.1 (-2.1, -0.1)* | 0.1 (-0.8, 1.0) | -1.2 (-2.5, 0.2) |
| **Waist-to-hip ratio** | Baseline | 1.0±0.1 | 1.0±0.1 |  |
|  | Changes after 6w. | 0.00 (-0.01, 0.02) | 0.00 (-0.01, 0.01) | 0.00 (-0.02, 0.02) |
|  | Changes after 12 w. | 0.00 (-0.01, 0.01) | 0.00 (-0.01, 0.02) | -0.01 (-0.02, 0.01) |

Values expressed as mean (SDs).  Mean differences (95% IC).  Comparisons between groups with one-way ANOVA and comparisons between groups after 6 and 12-weeks of intervention with unadjusted ANOVA. ^a^*P*: Significant differences (**P*<.05 or ***P*<.001) between before and after the intervention. BMI, body mass index; BW, body weight; DBP, diastolic blood pressure; HbA1c, glycated hemoglobin; HC, hip circumference; HDL: high-density lipoprotein; SBP, systolic blood pressure; LDL; low-density lipoprotein; w, week; WC, waist circumference.

Table 2. Changes in dietary intake of key foods at baseline and after 12- weeks of follow-up in the two study groups of the Greenhabit randomized controlled trial. This trial, conducted in Barcelona (Spain), evaluates the efficacy of a mHealth-based behavioral treatment for lifestyle modification in the self-management of type 2 diabetes.

|  |  | | **Greenhabit (n=50)** | | **Control (n=53)** | |  | |
| --- | --- | --- | --- | --- | --- | --- | --- | --- |
|  |  | Mean | | Mean | | *Differences between groups (Greenhabit vs control)* | |  |
|  |  |  | |  | |  | |  |
| **VOO, *g/d*** | Baseline | | 50.8±17.2 | | 49.3±13.7 | |  | |
|  | Changes after 12 w. | | 8.1 (3.2, 13.1)* | | 0.7 (-4.2, 5.6) | | 7.41 (0.46. 14.37) | |
| **Other oils, *g/d*** | Baseline | | 56.5±12.3 | | 54.4±12.6 | |  | |
|  | Changes after 12 w. | | 3.8 (-1, 8.6) | | -0.6 (-5.2, 3.9) | | 4.42 (-2.19. 11.03) | |
| **Total nuts, *g/d*** | Baseline | | 14.2±13.5 | | 14.0±13.0 | |  | |
|  | Changes after 12 w. | | 4.90 (1.31, 8.50)* | | 0.83 (-2.51, 4.16) | | 4.07 (-0.82. 8.97) | |
| **Vegetables, *g/d*** | Baseline | | 303.5±102.9 | | 302.8±106 | |  | |
|  | Changes after 12 w. | | -21.5 (-52.4, 9.3) | | -35.8 (-64, -7.6)* | | 14.3 (-27.52. 56.12) | |
| **Legumes, *g/d*** | Baseline | | 51.6±24.0 | | 57.5±26.8 | |  | |
|  | Changes after 12 w. | | 13.4 (0.98, 25.9)* | | -2.83 (-14.5, 8.9) | | 16.28 (-0.82. 33.38) | |
| **Fruits, *g/d*** | Baseline | | 289.5±159.5 | | 349.6±158.3 | |  | |
|  | Changes after 12 w. | | 4 (-39, 47) | | 3.4 (-37.6, 44.4) | | 0.57 (-58.81. 59.95) | |
| **Refined cereals, *g/d*** | Baseline | | 83.3±67.4 | | 86.5±66.1 | |  | |
|  | Changes after 12 w. | | -8.3 (-19.8, 3.1) | | 11.8 (0.9, 22.6)* | | -20.1 (-35.88. -4.31) | |
| **Whole grain cereals, *g/d*** | Baseline | | 26.7±41.0 | | 31.2±40.5 | |  | |
|  | Changes after 12 w. | | 2.27 (-6.72, 11.3) | | 1.55 (-7.0, 10.1) | | 0.72 (-11.68. 13.12) | |
| **Fish or seafood, *g/d*** | Baseline | | 81.8±32.2 | | 83.7±36.1 | |  | |
|  | Changes after 12 w. | | 0.6 (-7.7, 8.9) | | -0.2 (-7.7, 7.4) | | 0.73 (-10.49. 11.95) | |
| **Fat fish, *g/d*** | Baseline | | 16.8±14.7 | | 14.2±13.3 | |  | |
|  | Changes after 12 w. | | -0.08 (-3.95, 3.79) | | 1.30 (-2.33, 4.94) | | -1.38 (-6.69. 3.93) | |
| **Red meat*, g/d*** | Baseline | | 55.2±27.8 | | 52.6±42.9 | |  | |
|  | Changes after 12 w. | | 6.31 (-3.86, 16.50) | | -0.74 (-10.4, 8.92) | | 7.05 (-6.97. 21.08) | |
| **Processed meat, *g/d*** | Baseline | | 49.8±23.1 | | 43.0±33.5 | |  | |
|  | Changes after 12 w. | | 0.73 (-5.89, 7.35) | | -0.21 (-6.44, 6.02) | | 0.94 (-8.15. 10.03) | |
| **Pastries, cakes or sweets, *g/d*** | Baseline | | 30.8±25.7 | | 35.5±55 | |  | |
|  | Changes after 12 w. | | 4.8 (-3, 12.5) | | 7.9 (0.9, 14.9)* | | -3.15 (-13.57. 7.28) | |
| **Dairy products, *g/d*** | Baseline | | 408.4±230.2 | | 370.2±269.3 | |  | |
|  | Changes after 12 w. | | -5.80 (-52.7, 41.1) | | -8.22 (-52.3, 35.8) | | 2.42 (-61.91. 66.75) | |
| **Red wine, *g/d*** | Baseline | | 24.8±48.3 | | 33.3±67.5 | |  | |
|  | Changes after 12 w. | | -0.34 (-21.1, 20.4) | | 31.8 (12.5, 51.2)** | | -32.17 (-60.54. -3.8) | |
| **MedDiet P14-score, points** | Baseline | | 8.3±1.9 | | 8.4±2.1 | |  | |
|  | Changes after 6 w. | | -0.04 (-0.58, 0.50) | | -0.26 (-0.78, 0.25) | | 0.22 (-0.52. 0.97) | |
|  | Changes after 12 w. | | 0.37 (-0.15, 0.89) | | -0.02 (-0.52, 0.49) | | 0.39 (-0.34. 1.12) | |

Values expressed as mean (SDs).  Mean differences (95% IC).  Comparisons between groups with one-way ANOVA and comparisons between groups after 12-weeks of intervention with ANCOVA adjusted for baseline levels of each variable. d, day; VOO, virgin olive oil; w, week. Significant differences (**P*<.05 or ***P*<.001) between before and after the intervention. ƔP: Significant differences (*P*<.05) between-group changes.

Table 3. Changes in nutrient intake at baseline and after 12- weeks of follow-up in the two study groups of the Greenhabit randomized controlled trial. This trial, conducted in Barcelona (Spain), evaluates the efficacy of a mHealth-based behavioral treatment for lifestyle modification in the self-management of type 2 diabetes.

|  |  | **Greenhabit (n=50)** | **Control (n=53)** |  |
| --- | --- | --- | --- | --- |
|  |  | Mean | Mean | *Differences between groups (Greenhabit vs control)* |
|  |  |  |  |  |
| **Total energy, *kcal/d*** | Baseline | 2720±472.4 | 2682±727.2 |  |
|  | Changes after 12 w. | 101.9 (-13.1, 216.8) | 0.2 (-107.9, 108.3) | 101.64 (-56.18. 259.46) |
| **Carbohydrates, *g/d*** | Baseline | 211.4±51.6 | 217.8±81.5 |  |
|  | Changes after 12 w. | 5.9 (-6.4, 18.2) | 9.1 (-2.5, 20.7) | -3.19 (-20.09. 13.71) |
| **Protein*, g/d*** | Baseline | 119.4±22.5 | 116.7±30.5 |  |
|  | Changes after 12 w. | 4.0 (-1.5, 9.5) | -1.4 (-6.6, 3.7) | 5.44 (-2.07. 12.95) |
| **Total fat, *g/d*** | Baseline | 148.7±26.7 | 143.0±36.7 |  |
|  | Changes after 12 w. | 7.9 (0.04, 15.9)* | -5.4 (-12.8, 2.0) | 13.36 (2.49. 24.22) |
| **MUFA, *g/d*** | Baseline | 73.2±12.7 | 70.0±16.3 |  |
|  | Changes after 12 w. | 5.8 (1.5, 10.1)* | -1.7 (-5.8, 2.3) | 7.52 (1.62. 13.42) |
| **PUFA, *g/d*** | Baseline | 25.5±10.9 | 24.6±11.7 |  |
|  | Changes after 12 w. | 0.1 (-2.2, 2.4) | -1.7 (-3.9, 0.5) | 1.8 (-1.4. 5.01) |
| **SFA, *g/d*** | Baseline | 39.4±8.8 | 36.1±11.1 |  |
|  | Changes after 12 w. | 0.4 (-1.4, 2.3) | -0.1 (-1.9, 1.6) | 3.4 (-0.1. 6.9) |
| **Fiber, *g/d*** | Baseline | 31.5±8.1 | 33.5±10.3 |  |
|  | Changes after 12 w. | 1.3 (-1.1, 3.6) | 0.1 (-2.1, 2.3) | 1.16 (-2.06. 4.37) |
| **Vitamin A *mg/d*** | Baseline | 1600±699.1 | 1728±1263 |  |
|  | Changes after 12 w. | -48.4 (-180.7, 277.6) | -167.8 (-383.3, 47.7) | 119.35 (-195.21. 433.9) |
| **Vitamin B1*, mg/d*** | Baseline | 2.05±0.55 | 1.93±0.61 |  |
|  | Changes after 12 w. | 0.04 (-0.07, 0.16) | 0.01 (-0.10, 0.12) | 0.03 (-0.12. 0.19) |
| **Vitamin B9, μg*/d*** | Baseline | 485.5±112.3 | 489.2±135.0 |  |
|  | Changes after 12 w. | 8.80 (-23.4, 41.0) | 5.34 (-25.0, 35.6) | 3.46 (-40.74. 47.67) |
| **Vitamin B12, μg*/d*** | Baseline | 9.8±4.2 | 9.8±5.7 |  |
|  | Changes after 12 w. | -0.4 (-1.4, 0.7) | -0.6 (-1.6, 0.3) | 0.26 (-1.14. 1.65) |
| **Vitamin C, m*g/d*** | Baseline | 225.4±92.4 | 225.6±93.9 |  |
|  | Changes after 12 w. | 4.89 (-24.0, 33.8) | 28.3 (1.12, 55.5)* | -23.4 (-63.05. 16.25) |
| **Vitamin D, *μg/d*** | Baseline | 5.3±2.2 | 5.0±2.3 |  |
|  | Changes after 12 w. | 0.05 (-0.5, 0.6) | 0.2 (-0.3, 0.7) | -0.18 (-0.92. 0.56) |
| **Vitamin E, *μg/d*** | Baseline | 20.9±7.6 | 20.8±8.8 |  |
|  | Changes after 12 w. | -0.7 (-2.4, 1.0) | -1.1 (-2.7, 0.6) | 0.39 (-1.98. 2.75) |
| **Calcium, *mg/d*** | Baseline | 1132±341.9 | 1090±466.9 |  |
|  | Changes after 12 w. | 4.80 (-70.8, 80.4) | 10.6 (-60.5, 81.7) | -5.79 (-109.53. 97.94) |
| **Magnesium, *mg/d*** | Baseline | 459.7±105.0 | 457.6±125.4 |  |
|  | Changes after 12 w. | 17.4 (-6.55, 41.4) | 3.58 (-19.0, 26.1) | 13.86 (-19.07. 46.79) |
| **Iron, *mg/d*** | Baseline | 17.0±3.4 | 17.3±4.6 |  |
|  | Changes after 12 w. | 0.6 (-0.3, 1.5) | -0.1 (-1.0, 0.7) | 0.71 (-0.51. 1.94) |
| **Zinc, *mg/d*** | Baseline | 13.9±2.68 | 13.6±3.73 |  |
|  | Changes after 12 w. | 0.4 (-0.4, 1.1) | -0.5 (-1.1, 0.2) | 0.81 (-0.16. 1.77) |
| **Phosphorus, *mg/d*** | Baseline | 1909±376.1 | 1857±552.2 |  |
|  | Changes after 12 w. | 32.3 (-57.3, 121.8) | -18.4 (-102.7, 65.8) | 50.69 (-72.29. 173.67) |
| **Potassium, *mg/d*** | Baseline | 4765±971.3 | 4676±1161 |  |
|  | Changes after 12 w. | -9.31 (-264.6, 245.9) | -76.9 (-316.9, 163.2) | 67.56 (-282.87. 417.99) |
| **Sodium, *mg/d*** | Baseline | 4033±1123 | 3975±1369 |  |
|  | Changes after 12 w. | 0.8 (-216.4, 218.1) | 14.5 (-190.0, 218.8) | -13.61 (-311.85. 284.63) |
|  |  |  |  |  |

Values expressed as mean (SDs).  Mean differences (95% IC).  Comparisons between groups with one-way ANOVA and comparisons between groups after 12-weeks of intervention with ANCOVA adjusted for baseline levels of each variable. d, day; MUFA, monounsaturated fatty acid; PUFA, polyunsaturated fatty acid; SFA, saturated fatty acid; w, week. Significant differences (**P*<.05 or ***P*<.001) between before and after the intervention. ^Ɣ^P: Significant differences (*P*<.05) between-group changes.

Table 4. Changes of health-related quality of life at baseline and after 12-weeks of intervention in the two study groups of the Greenhabit randomized controlled trial. This trial, conducted in Barcelona (Spain), evaluates the efficacy of a mHealth-based behavioral treatment for lifestyle modification in the self-management of type 2 diabetes.

|  |  | **Greenhabit (n=50)** | **Control (n=53)** |  |  |
| --- | --- | --- | --- | --- | --- |
|  |  | Mean | Mean | *Differences between groups (Greenhabit vs control)* | *p-between groups* ^Ɣ^ |
|  |  |  |  |  |  |
| **LOT-R_expectations about life, *points*** | Baseline | 10.6±3.1 | 9.9±3.5 |  |  |
|  | Changes after 12 w. | 0.0 (-1.0, 0.9) | 0.3 (-0.7, 1.3) | -0.32 (-1.71. 1.06) | 0.64 |
| **H.A.D.S_depression, *points*** | Baseline | 17.5±1.3 | 17.0±1.6 |  |  |
|  | Changes after 12 w. | -0.1 (-0.6, 0.4) | 0.5 (-0.1, 1) | -0.56 (-1.34. 0.21) | 0.15 |
| **SF36_physical functioning** | Baseline | 64.4±21.2 | 70.3±16.4 |  |  |
|  | Changes after 12 w. | 2.7 (-0.5, 5.8) | 1.2 (-2, 4.3) | 1.51 (-2.95. 5.98) | 0.50 |
| **SF36_physical role** | Baseline | 89.5±28.5 | 97.1±12.5 |  |  |
|  | Changes after 12 w. | 1.2 (-1.8, 4.1) | 0.6 (-2.4, 3.6) | 0.58 (-3.62. 4.78) | 0.78 |
| **SF36_emotional role** | Baseline | 95.3±18.7 | 96.9±16 |  |  |
|  | Changes after 12 w. | -0.8 (-5.9, 4.4) | 2.3 (-2.8, 7.5) | -3.1 (-10.38. 4.18) | 0.40 |
| **SF36_mental health** | Baseline | 67.3±15.8 | 67.0±15.5 |  |  |
|  | Changes after 12 w. | -0.5 (-3.6, 2.7) | 2.4 (-0.7, 5.5) | -2.88 (-7.31. 1.54) | 0.20 |
| **SF36_general health** | Baseline | 53±19.2 | 55.9±19.8 |  |  |
|  | Changes after 12 w. | 2.4 (-1.1, 6) | 2.2 (-1.3, 5.7) | 0.23 (-4.75. 5.21) | 0.93 |
| **SF36_health change** | Baseline | 51.2±21.1 | 47.1±18.3 |  |  |
|  | Changes after 12 w. | 2.9 (-1.6, 7.5) | -1.2 (-5.7, 3.4) | 4.07 (-2.37. 10.51) | 0.21 |
| **Vigorous PA, *MET-minutes/week*** | Baseline | 1896±779.5 | 1120±277.1 |  |  |
|  | Changes after 12 w. | 0 (-303.3, 303.3) | 0 (-391.5, 391.5) | 0 (-495.22. 495.22) | 1.00 |
| **Moderate PA, *MET-minutes/week*** | Baseline | 1760±1466 | 2667±3582.8 |  |  |
|  | Changes after 12 w. | 560 (-542.7, 1663) | 213.3 (-423.3, 850) | 346.67 (-926.58. 1619.91) | 0.56 |
| **Time sitting, *minutes/day*** | Baseline | 382.1±599.4 | 344.7±175.7 |  |  |
|  | Changes after 12 w. | -95.5 (-244.6, 53.7) | 1.9 (-154.5, 158.4) | 97.39 (-118.75. 313.53) | 0.37 |
|  |  |  |  |  |  |

Values expressed as mean (SDs).  Mean differences (95% IC).  Comparisons between groups with one-way ANOVA and comparisons between groups after 12-weeks of intervention with ANCOVA adjusted for baseline levels of each variable MET, Metabolic equivalent of task; PA, physical activity; SF36, The Short Form-36 Health Survey. Significant differences (**P*<.05 or ***P*<.001) between before and after the intervention. ^Ɣ^*P*: Significant differences (*P*<.05) between-group changes.
